# Live Imaging at the Onset of Cortical Neurogenesis Reveals Differential Appearance of the Neuronal Phenotype in Apical versus Basal Progenitor Progeny

**DOI:** 10.1371/journal.pone.0002388

**Published:** 2008-06-11

**Authors:** Alessio Attardo, Federico Calegari, Wulf Haubensak, Michaela Wilsch-Bräuninger, Wieland B. Huttner

**Affiliations:** Max Planck Institute of Molecular Cell Biology and Genetics, Dresden, Germany; University of Washington, United States of America

## Abstract

The neurons of the mammalian brain are generated by progenitors dividing either at the apical surface of the ventricular zone (neuroepithelial and radial glial cells, collectively referred to as apical progenitors) or at its basal side (basal progenitors, also called intermediate progenitors). For apical progenitors, the orientation of the cleavage plane relative to their apical-basal axis is thought to be of critical importance for the fate of the daughter cells. For basal progenitors, the relationship between cell polarity, cleavage plane orientation and the fate of daughter cells is unknown. Here, we have investigated these issues at the very onset of cortical neurogenesis. To directly observe the generation of neurons from apical and basal progenitors, we established a novel transgenic mouse line in which membrane GFP is expressed from the beta-III-tubulin promoter, an early pan-neuronal marker, and crossed this line with a previously described knock-in line in which nuclear GFP is expressed from the Tis21 promoter, a pan-neurogenic progenitor marker. Mitotic Tis21-positive basal progenitors nearly always divided symmetrically, generating two neurons, but, in contrast to symmetrically dividing apical progenitors, lacked apical-basal polarity and showed a nearly randomized cleavage plane orientation. Moreover, the appearance of beta-III-tubulin–driven GFP fluorescence in basal progenitor-derived neurons, in contrast to that in apical progenitor-derived neurons, was so rapid that it suggested the initiation of the neuronal phenotype already in the progenitor. Our observations imply that (i) the loss of apical-basal polarity restricts neuronal progenitors to the symmetric mode of cell division, and that (ii) basal progenitors initiate the expression of neuronal phenotype already before mitosis, in contrast to apical progenitors.

## Introduction

The stem and progenitor cells that generate the neurons of the mammalian central nervous system (here collectively referred to as neural progenitors) build up a polarized tissue, the wall of the neural tube, whose inner side faces the lumen of the neural tube and whose outer side contacts the basal lamina. Initially in neural development, this wall consists of neuroepithelial cells, the primary neural progenitors, which form the pseudostratified neuroepithelium. With the onset and progression of neurogenesis, additional neural progenitors appear, and the neural tube wall adopts a multi-cell-layer architecture. Divisions of neural progenitors are confined to the innermost cell layer, the ventricular zone (VZ), and to the adjacent, telencephalon-specific, subventricular zone (SVZ) [1]–[5].

Neural progenitors in the developing cerebral cortex can be classified into two principal groups depending on whether they undergo mitosis at the apical or basal side of the VZ. Neuroepithelial cells and the related cells they transform into with neurogenesis, the radial glial cells [4], [6] and the recently described short neural precursors [7], [8], undergo mitosis at the lumenal surface, which corresponds to their apical side; these cells will therefore collectively be referred to as apical progenitors (APs). In addition to APs, previous work has established the existence neuronal progenitors that divide in the basal region of the VZ and in the SVZ; these progenitors have been called intermediate [5], [9], basal [4], [10] or SVZ [11] progenitors and will collectively be referred to as basal progenitors (BPs) here. Aside from the site of mitosis, APs and BPs can be distinguished by the expression of distinct transcription factors, i.e. Pax6 and Tbr2, respectively [12], [13].

A hallmark of APs, their apical-basal polarity, exists not only in interphase but is retained through mitosis. Specifically, mitotic APs exhibit distinct apical and basolateral plasma membrane and cortical domains [4], [14]–[18] and retain a basal process contacting the basal lamina [19], [20] (Y. Kosodo, K. Toida, V. Dubreuil, J. Schenk, E. Kiyokage, A. A., F. Mora-Bermudez, T. Arii and W. B. H., unpublished observations). This apical-basal polarity is thought to be of critical importance for the mode of division of APs, which can be either symmetric, with both daughter cells exhibiting the same fate, or asymmetric, with the daughters exhibiting different fates. On symmetric division, the cleavage plane is oriented parallel to the apical-basal cell axis and hence cell fate determinants with a polarized distribution in mitotic APs are equally distributed to the daughters, whereas on asymmetric division, this distribution is unequal because the cleavage plane orientation deviates from the apical-basal axis [4], [14], [21]. Importantly, APs, in particular in the telencephalon, are highly elongated cells, and hence their apical domain is relatively small as compared to their basolateral domain [14], [22]. Thus, the molecular machinery controlling mitotic spindle positioning and cleavage furrow ingression operates with remarkable precision in order to ensure cleavage parallel to the apical-basal cell axis, and down-regulation of this machinery is thought to result in asymmetric division by default [21].

Compared to APs, the mode of cell division of BPs is more restricted, with essentially all divisions being symmetric [9]–[11]. BPs arise from divisions of polarized APs and concomitant with, or following, the translocation of their cell body to the basal VZ or SVZ retract their apical process prior to mitosis [11]. In this context, given their origin and the symmetric mode of their cell division, a crucial question arises. Do mitotic BPs, despite this retraction, retain apical-basal polarity, as is the case for other delaminated mitotic progenitors, notably the paradigmatic Drosophila neuroblast [23]? Or have mitotic BPs, in contrast to other delaminated mitotic progenitors, lost apical-basal polarity?

Previously reported immunostainings for apical polarity markers have shown their concentration at the ventricular side of the VZ [16], [18], [24]–[26]. However, it should be stressed that these immunostainings are dominated by APs in interphase and allow no conclusion as to the question whether or not mitotic BPs, which at the onset of neurogenesis are a minor subpopulation relative to APs, have lost apical-basal polarity. Rather, answering this question requires that the analysis of mitotic BPs for the absence or presence of polarity markers is performed under conditions in which the entire shape of the dividing cell is revealed, which as far as we are aware has not been done.

Divisions of neural progenitors can generate either other progenitors, i.e. cells that re-enter the cell cycle, or neurons, i.e. post-mitotic cells. Cell cycle re-entry can be initiated rapidly after completion of cytokinesis, but this should not occur if a daughter cell is to become a neuron. A key question in neurogenesis therefore is when after progenitor division a neuronal phenotype, which is revealed by the appearance of a molecular marker specific of newborn neurons and indicative of a post-mitotic state, becomes apparent in the daughter cells. Moreover, it is unknown whether the time course of appearance of a neuronal phenotype is a stereotypical process, or differs between neurons arising from asymmetric *versus* symmetric divisions, or from APs *versus* BPs.

In the present study, we have used a novel approach that allowed us to address these two related questions at the very onset of neurogenesis. Most of the studies addressing the fate of APs and BPs by time-lapse imaging have been carried out at later stages of neurogenesis, due to the technical difficulties of examining these progenitors at the very onset of neurogenesis. Here, we explore the fate of AP and BP daughters at a developmental stage at which APs are still a relatively homogeneous population [8] and neuroepithelial rather than radial glial in nature [4], [6], and BPs begin to appear. To identify AP and BP divisions that generate neurons, and to study the cell polarity of mitotic neurogenic progenitors, we used a previously described knock-in mouse line in which GFP is expressed under the control of the promoter of *Tis21*, a gene specifically expressed in all progenitors undergoing neurogenic divisions [10], [27]. These comprise a small subpopulation of Pax6-positive APs and the vast majority of Tbr2-positive BPs [10], [13] (L. M. Farkas, C. Haffner, P. Khaitovich, K. Nowick, C. Birchmeier, S. Pääbo and W. B. Huttner, manuscript under revision). Moreover, we generated a novel transgenic mouse line, which allowed us to directly visualize, for the first time, using two-photon time-lapse video microscopy of neocortical slice cultures [10], the generation of neurons from neurogenic progenitors and the time course of appearance of a neuron-specific marker.

## Results

### The transgenic *Tubb3*-mGFP mouse line allows the identification of neurons in living tissue

To identify neurons generated by APs and BPs on live imaging, we generated a transgenic mouse line in which membrane-anchored GFP (mGFP) is expressed under the control of the promoter of the mouse *beta-III-tubulin* gene (*Tubb3*), which is specifically expressed in newborn neurons [28]. To this end, the sequence of GFP carrying the membrane anchor of GAP43 at its N-terminus [29], [30] was inserted, by RED-ET-based homologous recombination [31], [32], downstream of the start codon of the *Tubb3* gene (Fig. S1A), contained in a Bacterial Artificial Chromosome (BAC) that also included 50 kb-long 5′ and 3′ genomic flanking regions. The genotype of the resulting transgenic mouse line (Tg(*Tubb3*-GAP43-GFP)1Wbh, referred to as *Tubb3*-mGFP) was verified by Southern blot analysis (Fig. S1B).

**Figure 1:**
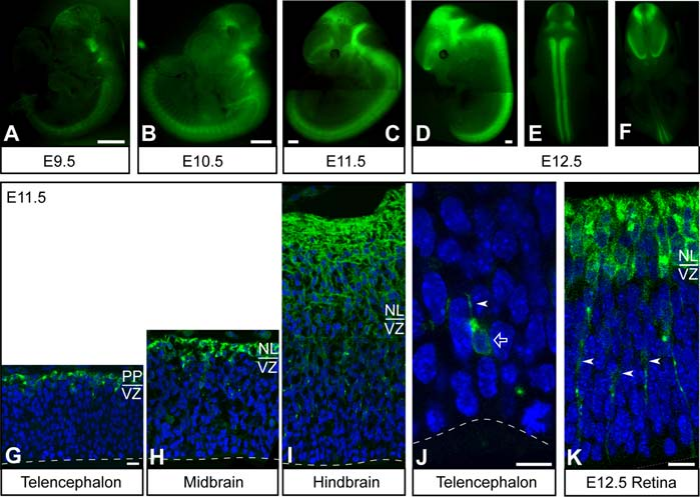
Intrinsic GFP fluorescence in the central nervous system of *Tubb3*-mGFP mouse embryos. (A–F) Whole-mount images of unfixed *Tubb3*-mGFP embryos at E9.5 (A), E10.5 (B), E11.5 (C) and E12.5 (D–F; E, dorsal view; F, ventral view). Background has been darkened electronically. Scale bars, 500 µm. (G–K) Confocal scanning photomicrographs (1-µm single optical sections) of 12 µm-thick cryosections through the E11.5 dorsal telencephalon (G, J), midbrain (H), hindbrain (I) and E12.5 retina (K) of *Tubb3*-mGFP mice. Green, intrinsic *Tubb3*-mGFP fluorescence; blue, Hoechst staining of nuclei. Note the thicker preplate (PP) and neuronal layers (NL) in the hindbrain than midbrain and telencephalon; VZ, ventricular zone. Ventricular (apical) surface is down (dashed lines). The arrow indicates a *Tubb3*-mGFP expressing cell (i.e., a neuron) whose soma is close to the ventricular surface; arrowheads indicate neuronal processes. Scale bars: G–I, 20 µm; J, K 10 µm.

We first investigated whether mGFP expression in heterozygous *Tubb3*-mGFP mice faithfully reproduced endogenous *Tubb3* expression temporally and spatially. GFP fluorescence could be detected in whole-mount embryos, postnatal neural tissue and fixed cryosections without antibody enhancement (Fig. 1 and Fig. S2). During embryonic development (E9.5–E14.5), the appearance and intensity of GFP fluorescence in the central and peripheral nervous system of *Tubb3*-mGFP mice precisely correlated with the known temporal and spatial gradients of neurogenesis (Fig. 1 and Fig. S2). Specifically, GFP fluorescence was detectable in E9.5 whole-mount embryos at the level of the spinal cord and hindbrain (with some axons projecting to the midbrain) (Fig. 1A). GFP fluorescence then progressively appeared rostrally and dorsally, i.e. in the midbrain, the ventral telencephalon and, finally, the dorsal telencephalon (E10.5–12.5, Fig. 1B–F). In the peripheral nervous system, GFP fluorescence was observed in the trigeminal ganglia, dorsal root ganglia and peripheral nerves (Fig. 1A–C and Fig. S2, A and B).

Similarly, at early postnatal (1 day) as well as adult (8 weeks) stages, GFP fluorescence in *Tubb3*-mGFP mouse whole-mounts was detected throughout the entire central nervous system (Fig. S2C and data not shown). However, in whole-mount brains of older animals (60 weeks), no GFP fluorescence was detected anymore, except for the olfactory bulb (data not shown). Given that the olfactory bulb is continuously replenished with newly generated neurons [33], [34], our observations imply that GFP fluorescence is lost with aging of neurons.

Analysis at the cellular level in fixed cryosections through the embryonic nervous system showed that, in the brain, *Tubb3*-mGFP fluorescence coincided with the neuronal layers (Fig. 1G–I). Occasionally, cells bearing a process that were mGFP-positive could be detected in the VZ (Fig. 1J). mGFP-positive cells were also found in other regions of the central and peripheral nervous system, such as the retina (Fig. 1K), spinal cord, dorsal root ganglia and peripheral nerves (Fig. S2, D and E). Importantly, mGFP fluorescence was not observed in the nucleus, consistent with the presence of a plasma membrane localization signal on the GFP (Fig. 1J).

**Figure 2:**
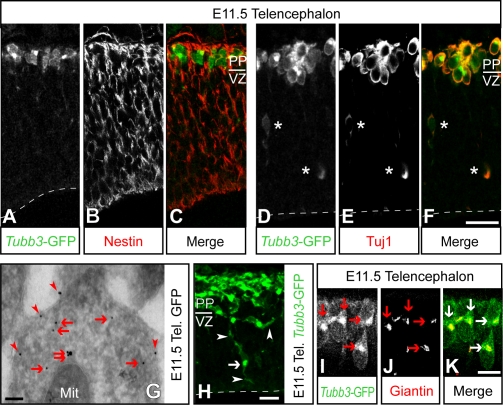
*Tubb3*-mGFP is specifically expressed in neurons and targeted to the Golgi complex and plasma membrane. (A–F and H–K) Confocal scanning photomicrographs (1-µm single optical sections; H, confocal stack) of 12–16 µm-thick cryosections through the E11.5 dorsal telencephalon of *Tubb3*-mGFP mice, showing intrinsic *Tubb3*-mGFP fluorescence (A, C green, D, F green, H, I, K green) and nestin (B, C red), Tuj1 (E, F red), or giantin (J, K red) immunofluorescence. Ventricular (apical) surface is down (dashed lines); PP, preplate; VZ, ventricular zone. Asterisks indicate neurons in the VZ. (G) GFP immunogold (10 nm) electron microscopy of a neuron in the dorsal telencephalon of an E11.5 *Tubb3*-mGFP mouse; Mit, mitochondrion. (G–K) Note the association of *Tubb3*-mGFP with the plasma membrane (arrowheads) and its concentration in the Golgi complex (arrows). Scale bars: A–F, 20 µm; G, 100 nm; H and I–K, 10 µm.

To corroborate that *Tubb3*-mGFP fluorescence was restricted to neurons and present throughout the entire neuronal population, immunohistochemistry was performed on cryosections through the E11.5 nervous system, using nestin as marker of neural progenitor cells [35], and beta-III-tubulin [28], MAP2 [36] and doublecortin [37] as markers of post-mitotic neurons (Fig. 2, A–F and S2, F–K). The cellular distribution of mGFP fluorescence was clearly distinct from that of nestin immunoreactivity (Fig. 2, A–C; Fig. S2, F–H) and overlapped that of beta-III-tubulin (Fig. 2, D–F), MAP2 (Fig. S2, I–K) and doublecortin (data not shown) immunoreactivity, indicating that *Tubb3*-mGFP is expressed in most, if not all, newborn neurons.

Immunogold electron microscopy showed that, at the subcellular level, *Tubb3*-mGFP immunoreactivity was associated with the plasma membrane (Fig. 2G, arrowheads) and with intracellular membrane structures (Fig. 2G, arrows). Analysis of the intrinsic *Tubb3*-mGFP fluorescence revealed, besides the plasma membrane (Fig. 2H, arrowheads), a strong signal in the perinuclear area (Fig. 2H, arrow). The latter reflected the Golgi complex, as confirmed by the colocalization with the immunostaining for giantin (Fig. 2, I–K), a Golgi complex marker [38]. Such clustering in the Golgi area has been observed previously [29] and reflects the palmitoylation and subsequent membrane insertion of the GAP43 plasma membrane localization signal. The membrane association of *Tubb3*-mGFP was confirmed by immunoblotting after sub cellular fractionation of brain homogenate, which showed that virtually all mGFP was recovered in a total membrane fraction (data not shown).

Taken together, we conclude that the *Tubb3*-mGFP transgenic mouse line allows the identification of newborn neurons by their intrinsic mGFP fluorescence at the systemic, tissue and cellular level in unfixed and fixed tissue.

### The *Tis21*-nucGFP/*Tubb3*-mGFP double transgenic mouse line as a novel tool to study progenitors generating neurons

**Figure 3:**
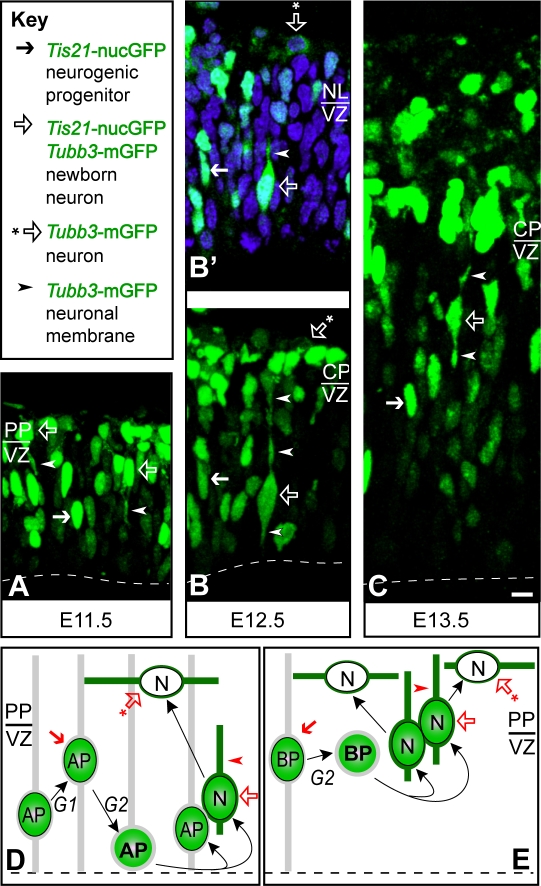
Distinct subcellular localization of nucGFP and mGFP allows distinction between neuronal progenitors and neurons in the double transgenic *Tis21*-nucGFP/*Tubb3*-mGFP mouse line. (A–C) Confocal scanning photomicrographs (stacks of 6–10 optical sections, 1-µm each) of 20 µm-thick cryosections through the dorsal telencephalon of E11.5 (A), E12.5 (B) and E13.5 (C) *Tis21*-nucGFP/*Tubb3*-mGFP double transgenic mouse embryos. (B') Single optical section (1 µm) of B. Green, intrinsic GFP fluorescence; blue, Hoechst staining of nuclei. Scale bar: A–C, 5 µm. (D and E) Cartoon illustrating presumptive cell lineages and the observed distinct subcellular localization of nucGFP *versus* mGFP; AP, apical progenitor; BP, basal progenitor; N, neuron; G1 and G2, phases of progenitor cell cycle. (A–E) Solid arrows, nuclei of neuronal progenitors showing *Tis21*-nucGFP fluorescence; open arrows, neuronal cell bodies showing *Tis21*-nucGFP plus *Tubb3*-mGFP fluorescence; open arrows with asterisk, neuronal cell bodies showing only *Tubb3*-mGFP fluorescence; arrowheads, neuronal processes showing *Tubb3*-mGFP fluorescence (see key in top left panel). Ventricular (apical) surface is down (dashed lines); PP, preplate; CP, cortical plate; VZ, ventricular zone.

To track neurogenic progenitors and identify their progeny as neurons, the transgenic *Tubb3-*mGFP mouse line, in which neurons are revealed by membrane-localized GFP, was crossed with the previously described *Tis21*-nucGFP knock-in mouse line [10], in which neurogenic progenitors are revealed by nucleoplasmic GFP, yielding the double transgenic mouse line here referred to as *Tis21*-nucGFP/*Tubb3*-mGFP (see Discussion for the reasons to choose one fluorescent protein in two subcellular locations). Importantly, the nuclear fluorescence of the *Tis21*-nucGFP and the neurite fluorescence of the *Tubb3-*mGFP were clearly distinguishable, allowing us to discern between cells that show either only one or both fluorescent cell compartments at the same time (Fig. 3). Analysis of cryosections of dorsal telencephalon at different stages of development (Fig. 3A–C) revealed the coexistence of three different cell populations, (i) cells only expressing *Tis21*-nucGFP, i.e. neurogenic progenitors (Fig. 3, solid arrows), (ii) cells only expressing *Tubb3*-mGFP, i.e. neurons (Fig. 3, open arrows with asterisk), and (iii) cells expressing *Tubb3*-mGFP that still showed *Tis21*-nucGFP fluorescence (Fig. 3, open arrows, arrowheads indicate mGFP-positive neurites). As *Tis21*-nucGFP is known to be inherited by newborn neurons and subsequently degraded [10], the cells showing both *Tubb3*-mGFP and *Tis21*-nucGFP fluorescence were newborn neurons, whereas the cells showing only *Tubb3*-mGFP fluorescence were older neurons (Fig. 3, D and E). Importantly, the temporal overlap of *Tis21*-nucGFP and *Tubb3*-mGFP fluorescence allowed us to directly observe the generation of neurons from APs (Fig. 3D) and BPs (Fig. 3E). This in turn set the stage to investigate, and compare, the neurogenic properties of APs and BPs.

To this end, we imaged mitotic *Tis21*-nucGFP-positive cells in slice cultures of *Tis21*-nucGFP/*Tubb3*-mGFP E10.5–12.5 dorsal telencephalon using two-photon time-lapse video microscopy, and monitored the behavior of the daughter cells arising from these divisions. Specifically, we analyzed APs and BPs for cleavage plane orientation, and their daughter cells for the onset of *Tubb3*-mGFP expression and residence of their nucleus in, *versus* exit from, the VZ, at the very onset of neurogenesis.

**Figure 4:**
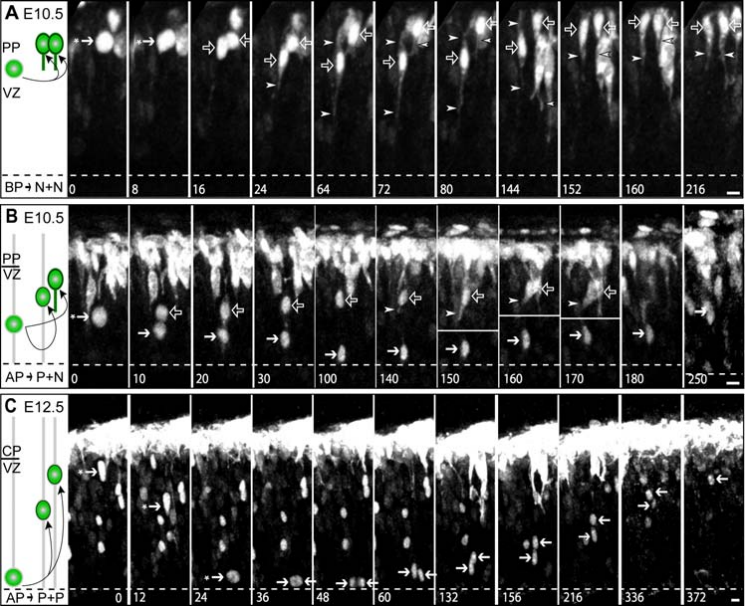
Two-photon imaging of apical and basal progenitor cell divisions and analysis of daughter cell fate in the double transgenic *Tis21*-nucGFP/*Tubb3*-mGFP mouse line. Two-photon time-lapse video microscopy of acute 500 µm-thick slice cultures prepared from dorsal telencephalon of E10.5 (A, B) and E12.5 (C) *Tis21*-nucGFP/*Tubb3*-mGFP double transgenic mouse embryos. (A) A neurogenic progenitor (solid arrows with asterisk), identified by *Tis21*-nucGFP expression, divides at the basal side of the ventricular zone (8–16 min). Both daughter cells (open arrows) are neurons as they show *Tubb3*-mGFP fluorescence in their processes (arrowheads), albeit with a different onset (left daughter, 24–216 min; right daughter, 72–216 min), and enter the neuronal layer (right daughter, 64–216 min; left daughter, 152–216 min). Note the transient apical migration of the left daughter (24–80 min). (B) A neurogenic progenitor (solid arrow with asterisk), identified by *Tis21*-nucGFP expression, divides in a subapical location (0–10 min). The nucleus of one of the daughter cells (open arrows) migrates rapidly towards the basal side (10–160 min) whereas that of the sibling daughter (solid arrows) migrates first to the apical surface (10–100 min) and then slowly basally (140–250 min). The cell with the leading nucleus shows *Tubb3*-mGFP fluorescence in its process (detectable as of 140 min, arrowheads), indicative of it being a neuron; this cell subsequently migrated perpendicular to the X-Y plane of imaging and could not be tracked further (170–250 min). The white lines at 150, 160 and 170 min were added to indicate that the upper and lower portions of the panels represent different sets of optical sections from the z-stack. Note that the cytoplasmic GFP fluorescence observed at 20 min reflects residual *Tis21*-nucGFP that is not yet re-sequestered into the daughter nuclei, rather than *Tubb3*-mGFP. (C) A neurogenic progenitor (solid arrows with asterisk), identified by *Tis21*-nucGFP expression, migrates rapidly from the basal boundary of the ventricular zone to the apical surface (0–24 min) and divides (24–48 min). The nuclei of the daughter cells (solid arrows) migrate towards the basal side (60–336 min), with one nucleus leading (right solid arrows). The leading nucleus was tracked until it entered the neuronal layer (after 372 min), whereas the trailing nucleus was tracked until it disappeared due to photobleaching (336–372 min). (A–C) Intrinsic GFP fluorescence is white. Each image is a maximum intensity projection of the single focal planes (2.7-µm steps) that showed the cells of interest. Numbers indicate tracking time (min). Time-lapse intervals: 8 min (A), 10 min (B), 12 min (C). Apical surface is down (dashed lines). Scale bars, 5 µm. The cartoons on the left summarize the observations; PP, preplate; CP, cortical plate; the boundary between the VZ and the preplate or cortical plate in the images is indicated by the line between VZ and PP or CP, respectively.

Representative examples of AP and BP divisions are shown in Fig. 4. Essentially all mitotic BPs at the onset of neurogenesis are positive for the marker Tbr2 (L. M. Farkas, C. Haffner, P. Khaitovich, K. Nowick, C. Birchmeier, S. Pääbo and W. B. Huttner, manuscript under revision). *Tis21*-nucGFP-positive neurogenic progenitors divided (i) at the basal side of the ventricular zone, generating two daughters which showed *Tubb3*-mGFP fluorescence (Fig 4A, left daughter 24 min, right daughter 72 min, arrowheads) and entered the neuronal layer; (ii) in a subapical location, generating two daughters of which only one showed *Tubb3*-mGFP fluorescence (Fig 4B, 140 min, arrowheads); or (iii) at the apical surface, generating two daughters which did not show *Tubb3*-mGFP fluorescence during the period in which tracking was possible (Fig 4C, 36–372 min).

### Basal *Tis21*-nucGFP–positive progenitors at the onset of neurogenesis divide mostly symmetrically with regard to daughter cell behaviour and generate neurons

**Figure 5:**
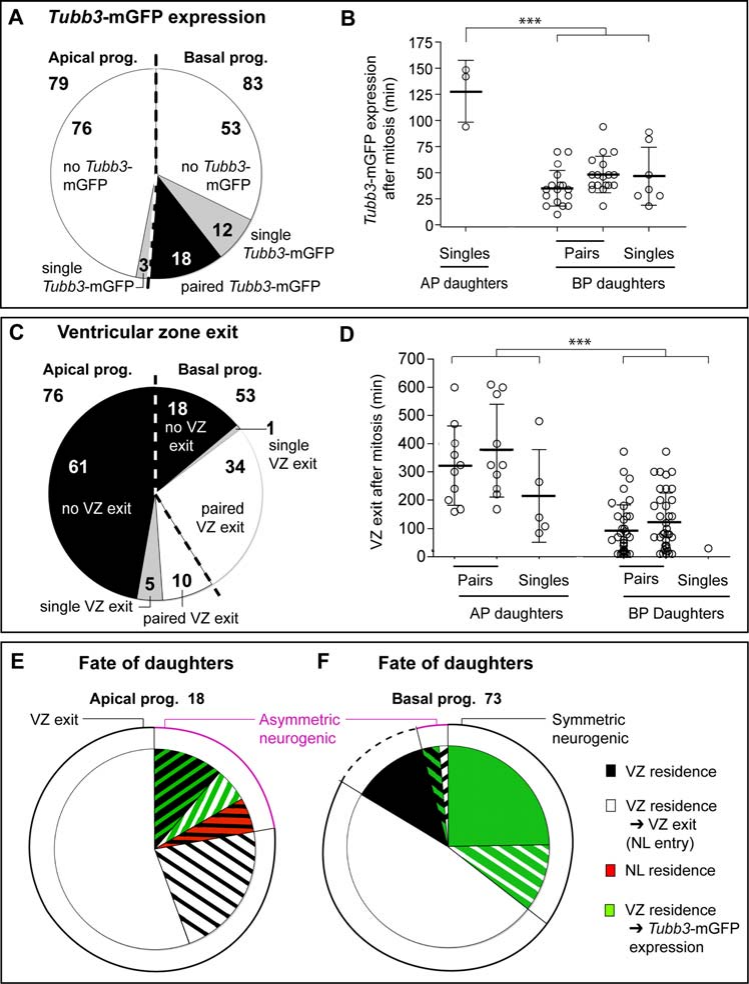
Behavior of daughter cells arising from apical and basal progenitors. Slice cultures prepared from dorsal telencephalon of E10.5–E12.5 *Tis21*-nucGFP/*Tubb3*-mGFP double transgenic mouse embryos were analyzed by two-photon time-lapse video microscopy for the behavior of daughter cells arising from *Tis21*-nucGFP–expressing APs (79 mitoses) and BPs (83 mitoses) in a total of 23 independent experiments. (A) Proportion of *Tis21*-nucGFP–positive AP and BP daughter cells that show discernible *Tubb3*-mGFP expression. Numerals in the sectors refer to the number of *Tis21*-nucGFP–expressing mother cells observed; white, no detectable (or discernible) *Tubb3*-mGFP expression in daughter cells; gray, discernible *Tubb3*-mGFP expression in one of the daughter cells; black, discernible *Tubb3*-mGFP expression in both daughter cells. (B) Onset of *Tubb3*-mGFP expression in daughter cells derived from *Tis21*-nucGFP–expressing APs and BPs. Open circles indicate individual daughters; bars, mean±S.D.; triple asterisk, p <0.001 (ANOVA) AP *versus* BP daughters. (C) Proportion of AP and BP daughter cells that show migration of *Tis21*-nucGFP–positive nuclei beyond the basal boundary of the ventricular zone (VZ exit). Only cases in which both daughter cells lacked *Tubb3*-mGFP expression were scored. Numerals in the sectors refer to the number of *Tis21*-nucGFP–expressing mother cells observed; black, both daughter cell nuclei remain in the VZ; gray, VZ exit of one daughter cell nucleus; white, VZ exit of both daughter cell nuclei. (D) Time point of VZ exit of the *Tis21*-nucGFP–positive nucleus of *Tubb3*-mGFP–negative daughter cells derived from APs and BPs. Note that for single daughters exiting the VZ, only cases in which the other daughter cell lacked *Tubb3*-mGFP expression were scored. Open circles indicate individual daughters; bars, mean±S.D.; triple asterisk, p <0.0001 (t-test) AP *versus* BP daughters. (E and F) Fate of daughter cells arising from APs (E, 18 cases) and BPs (F, 73 cases). Width of the sectors reflects the number of daughter cell pairs observed. The circle lines indicate the major classes of behavior of daughter cell pairs. (E) Green/black, one daughter expresses *Tubb3*-mGFP and one daughter nucleus remains in the VZ; green/white, one daughter expresses *Tubb3*-mGFP and one daughter nucleus exits the VZ; black/red, one daughter nucleus remains in the VZ and one exits and can be tracked in the NL, with the time of observation exceeding the mean length of one cell cycle [55]; black/white, one daughter nucleus remains in the VZ and one exits; solid white, both daughter nuclei exit the VZ. (F) Solid green, both daughters express *Tubb3*-mGFP; green/white, one daughter expresses *Tubb3*-mGFP and one daughter nucleus exits the VZ; solid white, both daughter nuclei exit the VZ; solid black, both daughter nuclei remain in the VZ longer than 100 min; green/black, one daughter expresses *Tubb3*-mGFP and one daughter nucleus remains in the VZ; white/black, one daughter nucleus exits, and the other remains in, the VZ.

In 30 of 83 cases, the *Tis21*-nucGFP-positive BP daughters rapidly showed *Tubb3*-mGFP fluorescence (Fig. 5A and F, green sectors; Fig. S3B, green bars). In 18 of these 30 cases, *Tubb3*-mGFP expression was detectable in both of the daughters (Fig. 4A and Movie S1, Fig. 5A), with an essentially identical onset (Fig. 5B). When *Tubb3*-mGFP expression was discernible in only one of the BP daughters (12 of 30 cases), it showed the same onset as when it was detectable in both (Fig. 5B). The reason why appearance of *Tubb3*-mGFP fluorescence was not observed in the other daughter in these 12 cases, and in neither daughter in 34 other cases (see below), was mostly entry into, and residence in, the neuronal layer (Fig. S3B, white and red bars, respectively; average tracking 227 min, max 900 min), upon which discerning *Tubb3*-mGFP expression became very difficult due to the presence of neighboring *Tubb3*-mGFP–positive cells. Other reasons included photobleaching while still in the VZ, or migration out of the focal plane (Fig. S3B, black bars, see below).

In 53 of 83 cases, *Tubb3*-mGFP expression was not detected in BP daughter cells while their nucleus was within the VZ. We reasoned that exit from the VZ, especially at this early developmental stage at which the cortical plate is beginning to form, is strongly suggestive of a neuronal fate, given that the vast majority of BP daughters that have exited the VZ have been reported to not return to the VZ and to be postmitotic [9]. We then studied the proportion and the timing of VZ exit of BP daughters in our time-lapse experiments.

In 35 of these 53 cases, nuclei could be followed exiting the VZ and residing in the forming cortical plate, which occurred almost always symmetrically, i.e. for both daughters (34 of 35) (Fig. 5C and F solid white sector; Fig. S3B, white and red bars, respectively), thus suggesting a neuronal fate for both them. We therefore conclude that the majority (64 of 83) of divisions of *Tis21*-nucGFP–positive BPs are symmetric with regard to daughter cell behavior (Fig. 5F, solid black circle line), and show an overt (*Tubb3*-mGFP expression; Fig. 5F, solid green and green-white sectors) or highly probable (entry and residence in neuronal layer; Fig. 5F, solid white sector) neuronal fate.

In 18 of the 53 cases of BP daughter cells in which *Tubb3*-mGFP expression was not detected within the VZ, the track of both nuclei was lost in the VZ (Fig. 5C; Fig. S3B, black bars). Here, we distinguished between the cases in which this occurred within, *versus* after, the first 100 min (Fig. S3B, blue line). The value of 100 min corresponds to the mean VZ residence time of those BP daughter nuclei (or cell bodies) that were seen to eventually enter the neuronal layer. When both nuclei could be tracked in the VZ for longer than 100 min (9 of 18), we considered that the lack of entry into the neuronal layer might be significant, perhaps indicating a delayed onset of the neuronal phenotype. We therefore included only these 9 out of 18 cases in our analysis (Fig. 5F, solid black sector; Fig S3B vertical black lines).

### Apical *Tis21*-nucGFP–positive progenitors generate daughters that do not express *Tubb3*-mGFP while being in the VZ

Surprisingly, in the case of *Tis21*-nucGFP-positive AP daughters, *Tubb3*-mGFP expression could be detected in only the minority of cases (3 of 79, Fig. 5A and E, green-black and green-white sectors). When daughters of *Tis21*-nucGFP-positive APs showed *Tubb3*-mGFP expression, this occurred exclusively in one of the daughters (Fig. S3A, green bars), which always was the more basally located daughter (Fig. 4B, open arrows; Movie S2). In the vast majority of cases (76 of 79), however, an induction of *Tubb3*-mGFP expression in AP daughters was not observed while these were in the VZ, although tracking was performed on average for 282 min and for as long as 804 min (Fig. 5E, red-black and white-black sectors). Reasons why tracking of *Tis21*-nucGFP-positive nuclei became impossible included photobleaching, migration out of the focal plane and proximity to other *Tis21*-nucGFP-positive cells (Fig. S3A, black bars). It should be noted that nuclei pairs whose tracking ended in the VZ (61 of 76 cases; Fig. 5C and Fig. S3A, black bars, 248±140 min) were always engaged in apical-to-basal migration. In 15 of 76 cases (Fig 5C), the track of one or both daughter nuclei could be followed until they entered the neuronal layer (Fig. 4C and Movie S3; Fig S3A, white bars, 329±156 min), upon which discerning a possible *Tubb3*-mGFP expression became very difficult due to the presence of neighboring *Tubb3*-mGFP–positive cells. The behavior of AP daughter pairs of which at least one showed either *Tubb3*-mGFP expression or entry into the neuronal layer is summarized in Fig. 5E (the cells included in this analysis are indicated by vertical black lines in Fig. S3A). We conclude that only a minority of *Tis21*-nucGFP-positive AP daughters show an overt neuronal fate while migrating through the VZ, and that the majority are either neurons whose onset of *Tubb3*-mGFP expression is delayed until entry into the neuronal layer, or BPs (see Discussion).

### Comparison of apical *versus* basal progenitor daughter behavior

Apical and basal progenitor daughters differed not only with regard to the frequency with which *Tubb3*-mGFP expression was detected, but also with regard to the onset of *Tubb3*-mGFP expression. Specifically, *Tubb3*-mGFP fluorescence appeared much faster in BP daughters (45±23 min, n = 48; earliest appearance after 12 min) than AP daughters (127±27 min, n = 3) (Fig. 5B).

Differences between BP and AP daughters were also evident regarding the pattern of nuclear (or cell body) migration in the VZ. BP daughter pair nuclei exhibited different patterns of migrations, showing either (i) no significant migration (because the daughter cells were already born at the boundary between VZ and neuronal layer; 6 of 82 cases); (ii) basal migration with the same mean speed (57 of 82 cases); or (iii) basal migration with different mean speed (19 of 82 cases); the latter included cases of (iv) transient migration for up to 12 µm towards the apical side (5 of 82 cases; for an example, see Fig. 4A, left open arrows, and Movie S1). In contrast, in all cases observed, the nuclei of AP daughters migrated towards the basal side of the VZ with one sibling preceding the other. Sibling nuclei migrated either with the same mean speed (41 of 79 cases; for an example, see Fig. 4C and Movie S3) or with different mean speed (38 of 79 cases; for an example, see Fig. 4B and Movie S2), as previously reported [9], [10].

### Increased randomization of cleavage plane orientation in basal as compared to apical *Tis21*-nucGFP–positive progenitors

**Figure 6:**
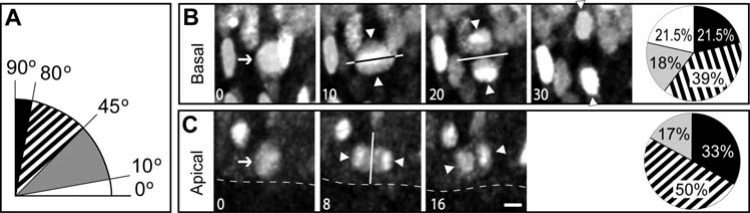
Cleavage plane orientation in apical and basal progenitors. Slice cultures prepared from dorsal telencephalon of E10.5-E12.5 *Tis21*-nucGFP/*Tubb3*-mGFP double transgenic mouse embryos were analyzed by two-photon video microscopy for the cleavage plane orientation of mitotic APs and BPs. (A) Diagram showing the four classes of cleavage planes; 0° corresponds to parallel (horizontal cleavage plane), and 90° to perpendicular (vertical cleavage plane), to the ventricular surface (dashed lines in (C). (B, C) Examples of a horizontal cleavage plane (solid lines) in a BP (B, arrow) and a vertical cleavage plane (solid lines) in an AP (C, arrow); time intervals were 10 min (B) and 8 min (C) (see numbers in the lower left corner of the panels). Pie charts show the distribution of cleavage plane orientation between the four classes defined in (A) for 33 mitoses of BPs (B) and 42 APs (C). Only cases in which cytoplasmic *Tis21*-nucGFP could be observed (i.e. the cell being in anaphase; B, 10 min; C, 8 min) and which therefore allowed the deduction of cleavage plane orientation were included (75 of 184); white triangles indicate the position of daughter cell chromosomes/nuclei. Scale bar, 5 µm.

Previous studies on cleavage plane orientation in APs have shown that the switch from symmetric to asymmetric division can be caused by a deviation of the cleavage plane from the prevailing orientation parallel to the apical-basal axis of the VZ [4], [14], [21], [39]–[41]. In light of the observation that *Tis21*-nucGFP–positive BPs divided almost always symmetrically (in that both daughters exhibited a phenotype characteristic of a neuronal fate) (Fig. 5B inset), it became important to investigate cleavage plane orientation in this type of progenitor and to compare it with that of APs. We investigated this issue on live imaging that yielded three-dimensional information of progenitor cell division, exploiting the fact that, as a result of nuclear envelope break-down during prophase, *Tis21*-nucGFP diffuses into the cytoplasm, which in turn reveals the ingression of the cleavage furrow during cytokinesis. This, together with the initial position of the reforming nascent daughter cell nuclei in telophase, revealed by re-entry of *Tis21*-nucGFP into the nucleus, allowed the determination of cleavage plane orientation. We arbitrarily defined four groups of cleavage plane orientation relative to the ventricular surface (Fig. 6A).

The vast majority (>80%) of *Tis21*-nucGFP–positive APs divided with a vertical (90-80°; for an example, see Fig. 6C) or vertical-oblique (80-45°) cleavage plane orientation; no horizontal (10-0°) and only a small proportion of horizontal-oblique (45-10°) cleavage planes were observed (Fig. 6C). Cleavage planes in *Tis21*-nucGFP–positive BPs did not show a preference for vertical or vertical-oblique orientations but rather a trend towards a randomized orientation (Fig. 6B), with a substantial proportion of horizontal cleavage planes (for an example, see Fig. 6B).

### *Tis21*-nucGFP–positive mitotic basal progenitors lack apical-basal polarity

In the case of APs, the orientation of the cleavage plane is significant because it is related to the apical-basal axis of these highly polarized cells and determines whether polarized cell fate determinants are distributed equally or unequally to the daughter cells, and hence whether their fate is symmetric or asymmetric [4], [14], [21], [39]–[41]. Symmetric divisions of APs are thought to require a perfect alignment of the cleavage plane parallel to their apical-basal axis, whereas deviation of the cleavage plane from this axis is sufficient for asymmetric division to occur [14], [21], [22]. Therefore, our observations that neurogenic BPs divide with nearly randomized cleavage plane orientation (Fig. 6B) but yield daughter cells with predominantly symmetric fate (Fig. 5F) are paradoxical, unless mitotic BPs were to lack apical-basal polarity.

**Figure 7:**
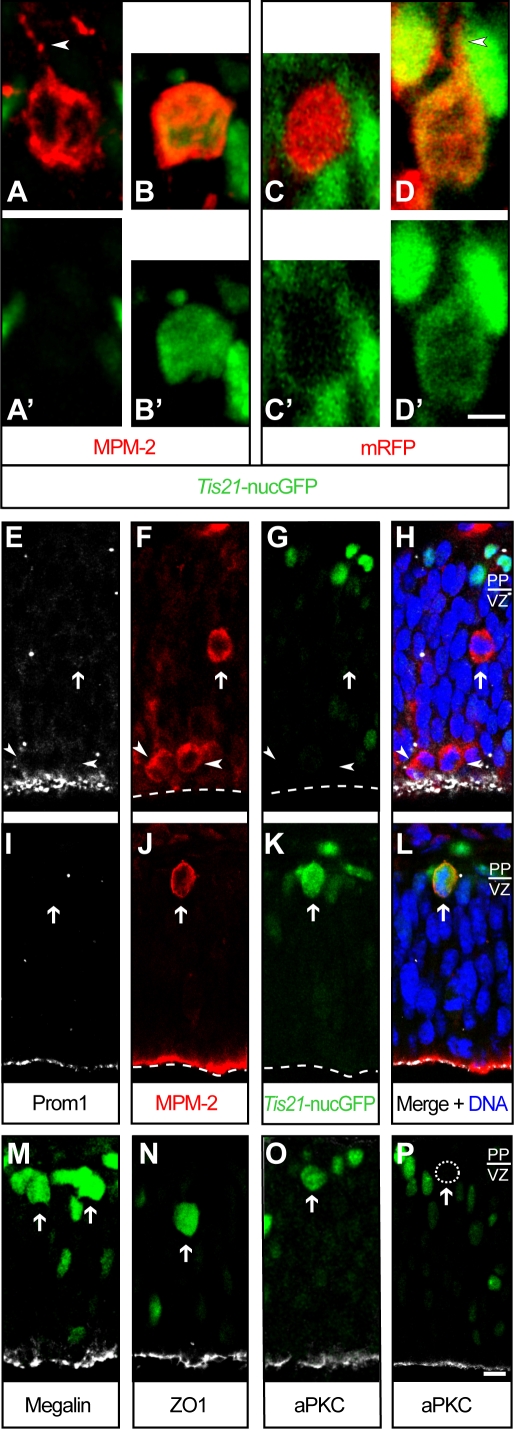
Lack of apical-basal polarity in mitotic basal progenitors. Confocal scanning photomicrographs of 20 µm-thick fixed cryosections through the dorsal telencephalon of heterozygous E10.5 (A–B'), E13.5 (C–D'), E10.5 (E–H) and E11.5 (I–L) *Tis21*-nucGFP knock-in mouse embryos. (A–D') Stack of 7–10 optical sections (1 µm each) showing *Tis21*-nucGFP–negative (A, A', C, C') and *Tis21*-nucGFP–positive (B, B', D, D') mitotic BPs (identified by Hoechst staining and phosphohistone 3 immunofluorescence, not shown); red, MPM-2 immunofluorescence (A, B) or intrinsic mRFP fluorescence after *in utero* electroporation at E12.5 (C, D); green, intrinsic GFP fluorescence (A–D'). Note the absence of apically-directed processes in both *Tis21*-nucGFP–negative and –positive mitotic BPs. Arrowheads, short basal extension from the cell body that did not exceed one cell body diameter. Scale bar in D', 5 µm. (E–P) Single optical sections (1 µm) showing *Tis21*-nucGFP–negative (E–H, P) and *Tis21*-nucGFP–positive (I–O) mitotic BPs (arrows) and APs (arrowheads); white, prominin-1 (E, H, I, L), megalin (M), ZO-1 (N) or aPKC (O–P) immunofluorescence; red, MPM-2 immunofluorescence (F, H, J, L); green, intrinsic GFP fluorescence (G, H, K, L, M–P); blue, Hoechst staining (H, L). Note the absence of prominin-1, megalin, ZO-1 and aPKC immunoreactivity in both *Tis21*-nucGFP–negative and –positive mitotic BPs, but its presence in mitotic APs and on the apical surface. The prominin-1 immunoreactive dots in the VZ (E–H, I–L) are extracellular particles known to be present within the neuroepithelium [44], [45]; note that these are spatially distinct from the mitotic BPs. Dashed lines, apical plasma membrane; dashed circle in (P) outlines the soma of a *Tis21*-nucGFP–negative mitotic BP (as determined by Hoechst staining, data not shown). PP, preplate. Scale bar in P, 10 µm. (A–P) Ventricular (apical) surface is down.

To investigate this issue, we first examined, from the onset of neurogenesis to mid-neurogenesis, *Tis21*-nucGFP–positive mitotic BPs for the possible presence of an apical process contacting the ventricular surface. For this purpose, we performed confocal microscopy on cryosections of E10.5–11.5 dorsal telencephalon labeled with MPM-2 antibody, which stains the cytoplasm of mitotic cells [42] including their processes (Y. Kosodo, K. Toida, V. Dubreuil, J. Schenk, E. Kiyokage, A. A., F. Mora-Bermudez, T. Arii and W. B. H., unpublished observations), and of E13.5 dorsal telencephalon after *in utero* electroporation of monomeric red fluorescent protein (mRFP) [43], a reporter protein that freely diffuses in the cytoplasm. This revealed that *Tis21*-nucGFP–positive mitotic BPs lacked an apical process that extended to the ventricular surface (Fig. 7, B, B', D, D'; Table 1 left, rows *Tis21*-nucGFP +). In fact, the majority of *Tis21*-nucGFP–positive mitotic BPs (49 of 71) showed a total lack of an apically directed process, and the remainder (22 of 71) showed a short (≤5 µm) apically directed process remnant (Table 1 left, rows *Tis21*-nucGFP +).

**Table 1:** Lack of ventricular contact and apical markers in mitotic basal progenitors.

Age	Cell shape marker	*Tis21*-nuc GFP	Cells	Ventricular contact		Short extensions from cell body				Apical marker expression		Apical marker
						apical		basal				
				+	–	+	–	+	–	+	–	
**E13.5**	**mRFP**	+	6	0	6	2	4	2	4	n.d.	n.d.	
		–	5	0	5	0	5	0	5	n.d.	n.d.	
**E11.5**	**MPM-2**	+	13	0	13	3	10	2	11	n.d.	n.d.	
		–	10	0	10	1	9	3	7	n.d.	n.d.	
**E10.5**	**MPM-2**	+	16	0	16	5	11	3	13	2*	14	**Prom1**
		–	2	0	2	0	2	1	1	1*	1	
**E10.5**	**MPM-2**	+	14	0	14	5	9	3	11	0	14	**Megalin**
		–	6	0	6	1	5	1	5	0	6	
**E10.5**	**MPM-2**	+	22	0	22	7	15	2	17	0	22	**ZO-1**
		–	5	0	5	1	4	0	5	0	5	
**E13.5**	**Membrane GFP**		9	0	9	0	9	0	9	n.d.	n.d.	
**Subtotal (mRFP, MPM-2)**		+	71	0	71	22	49	12	59	2	50	
		–	28	0	28	3	25	5	23	1	12	
**Total**			108	0	108	25	83	17	91	3	62	

Heterozygous *Tis21*-nucGFP embryos were analyzed, with or without *in utero* electroporation of either mRFP or membrane GFP at E12.5 followed by expression for 24 h as indicated. Analysis of mitotic BPs in the dorsal telencephalon was carried out by two-photon video microscopy of slice cultures (membrane GFP) or by confocal microscopy of 20-µm fixed cryosections of intrinsic mRFP fluorescence or double immunofluorescence for MPM-2 and either prominin-1 (Prom1), megalin or ZO-1 as indicated. The latter data are from three littermate embryos, each immunostained for each marker on at least 5 cryosections. Mitotic BPs were scored for the absence (–) or presence (+) of *Tis21*-nucGFP fluorescence, mRFP–, GFP– or MPM-2–stained apical processes contacting the ventricle or processes extending apically and basally from the cell body, and polarized immunoreactivity of apical markers. Note that any apical and basal extensions from the cell body did not exceed one cell body diameter in length. Asterisks indicate prominin-1 immunoreactivity over the cell body due to extracellular particles known to be present within the neuroepithelium [44], [45]; n.d., not determined.

We next investigated whether *Tis21*-nucGFP–positive mitotic BPs, despite the lack of an apical process contacting the ventricular surface, retain apical-basal polarity, by examining the possible presence of markers characteristic of the apical pole of the cell. Cryosections of E10.5 dorsal telencephalon were double-immunostained with MPM-2 antibody and for either (i) prominin-1 (CD133), a pentaspan membrane protein localized in protrusions of the apical plasma membrane of neuroepithelial cells [25], [44], [45]; (ii) megalin, an integral membrane protein found on the apical plasma membrane and in apical endosomes of neuroepithelial cells [45], [46]; (iii) ZO-1, a peripheral membrane protein concentrated at apical cell junctions of neuroepithelial cells [24]; or (iv) atypical protein kinase C (aPKC), a constituent of the Par protein complex which in neuroepithelial cells is known to be associated with the apical cell cortex [26].

Confocal microscopy revealed that *Tis21*-nucGFP–positive mitotic BPs, in contrast to mitotic APs, lacked these markers (Fig. 7E–P; Table 1 right, rows *Tis21*-nucGFP +). Prominin-1 and aPKC immunoreactivity was essentially undetectable, and megalin and ZO-1 immunoreactivity, when detectable, was drastically reduced and, importantly, non-polarized. These observations indicate that *Tis21*-nucGFP–positive mitotic BPs, in contrast to mitotic APs, do not exhibit apical-basal polarity.

### *Tis21*-nucGFP–negative mitotic basal progenitors also lack apical-basal polarity

*Tis21*-nucGFP–positive mitotic BPs constitute the vast majority, but not all, of the basally dividing cells in the rodent dorsal telencephalon [10]. As a minor proportion of basally dividing cells in the rodent dorsal telencephalon has been reported to generate progeny that divides again [5], [9], [47], it was of interest to investigate whether *Tis21*-nucGFP–negative mitotic BPs might, perhaps, retain certain features of apical-basal polarity. However, using the same experimental approach as for *Tis21*-nucGFP–positive mitotic BPs, neither an apical process contacting the ventricular surface (Fig. 7, A, A', C, C'; Table 1 left, rows *Tis21*-nucGFP –) nor prominin-1 (Fig. 7E–H) or significant megalin, ZO-1 (data not shown) and aPKC (Fig. 7P) immunoreactivity were detected in *Tis21*-nucGFP–negative mitotic BPs (Table 1 right, rows *Tis21*-nucGFP –). The proportion of *Tis21*-nucGFP–negative mitotic BPs showing a short apically directed process remnant was less (3 of 28, i.e. 11%) than for the *Tis21*-nucGFP–positive mitotic BPs (22 of 71, i.e. 31%; see Table 1 left).

To corroborate that BPs, during mitosis, lack an apical process contacting the ventricular surface, we searched for the possible existence of cellular processes by using a general plasma membrane marker. Dorsal telencephalon of E12.5 *Tis21*-nucGFP embryos was electroporated *in utero* with mGFP under the control of a constitutive promoter, and organotypic slice cultures were analyzed by two-photon time-lapse video microscopy. This confirmed the lack of apical contact of progenitors dividing at the basal side of the VZ (Table 1). In addition, this approach revealed the retraction of the apical process prior to mitosis (Movie S4), confirming previous observations [11] and a report [48] that appeared after submission of the present study. In conclusion, all mitotic BPs, irrespective of *Tis21*-nucGFP expression, lack apical-basal polarity.

## Discussion

We have investigated BPs, an intermediate in the lineage from neuroepithelial and radial glial cells to neurons [4], [5], [9]–[11], for the relationship between apical-basal polarity, cleavage plane orientation, the mode of cell division and the fate of the daughter cells originating from their divisions. Moreover, we compared BPs with APs, for which this relationship has been extensively studied [4], [14], [17], [21], [22], [24], [39]–[41], [49]–[51], at the onset of neurogenesis when APs are thought to be still relatively homogeneous [8], and focused especially on the appearance of the neuronal phenotype as revealed by *Tubb3*-mGFP expression, and its kinetics. For this purpose, we developed a novel approach to simultaneously identify neurogenic progenitors and their progeny on live imaging.

### A novel approach to visualize neurogenic progenitors generating neurons on live imaging

We studied progenitor cell division and the behavior of the daughter cells in slice cultures of dorsal telencephalon using two-photon excitation, which allowed us to perform live imaging deep into tissue (up to 200 µm) and hence to follow a population of cells. To be able to simultaneously observe both, neurogenic progenitors and neurons, we crossed the previously described *Tis21*-nucGFP knock-in line, in which nuclear GFP is selectively expressed in all neurogenic progenitors [10], with a newly generated BAC-transgenic line expressing a fluorescent reporter protein under the control of the early pan-neuronal *beta-III-tubulin* promoter (*Tubb3*), i.e. in all newborn neurons irrespective of the specific neuronal subtype. It is important to emphasize that the present two-photon microscopy set-up performs best with GFP. We therefore used also GFP for the *Tubb3* transgenic line, but targeted it to a subcellular location distinct from *Tis21*-nucGFP, i.e. the Golgi complex/plasma membrane (*Tubb3*-mGFP), to be able to discern progenitor-expressed and neuron-expressed GFP. This allowed us to obtain dual information with one fluorescent protein, and to directly (i.e. without the need of post-imaging identification) observe – as far as we are aware, for the first time – a progenitor cell generate a neuron.

With the present approach of using the same fluorescent protein in two distinct subcellular locations, we could, on live imaging, readily distinguish (i) cells showing only nuclear fluorescence (e.g., Fig. 4C, Movie S3), (ii) cells showing both nuclear and plasma membrane fluorescence (e.g., Fig. 4A, Movie S1), and (iii) cells lacking nuclear but showing plasma membrane (and Golgi) fluorescence (e.g., Fig. S3A, Movie S5). It should be noted that the transient presence of *Tis21*-nucGFP in the cytoplasm during mitosis due to nuclear envelope breakdown did not lead to false-positive staining of neurites because the *Tis21*-nucGFP was confined to the cell body cytoplasm and was re-sequestered into the nucleus by the end of telophase.

Moreover, besides being able to observe early neurogenesis in intact tissue, the association of the GFP with specific subcellular structures made it possible to also study cell biological features relevant in this context. With regard to APs and BPs, the orientation of the cleavage plane could be deduced from the *Tis21*-nucGFP fluorescence, which outlined the cleavage furrow and revealed the position of the nascent daughter cell nuclei. With regard to the neuronal daughters generated, the *Tubb3*-mGFP fluorescence revealed early events of neuronal polarization such as Golgi complex positioning and neurite outgrowth, as is described in the Supplemental Data (Text S1, Figure S4 and Movies S5 and S6).

### Symmetric neurogenic divisions of basal progenitors and the rapid appearance of neuronal properties

Our observations on the behavior of the daughter cells arising from divisions of *Tis21*-nucGFP–positive, i.e. neurogenic, BPs strongly suggest that almost all of these divisions (96%) are symmetric, i.e. generate daughters with similar behavior and fate. Our data extend previous conclusions in this regard [9]–[11] to the very onset of neurogenesis. In fact, the majority of BP daughters (86%) behaved like neurons, either showing *Tubb3*-mGFP fluorescence (33%) or entering the neuronal layer (53%) and, when the cell could be tracked (25%), remaining in it. However, we cannot exclude that some of the *Tis21*-nucGFP–positive BP-derived daughters for which we were unable to observe *Tubb3*-mGFP expression underwent cell division, i.e. were progenitors rather than neurons. In addition, we did observe a small proportion (14%) of daughter cells that remained in the VZ for longer (>100 min) than the mean VZ residence time of those daughters that entered the neuronal layer. Given that also these daughters originated from *Tis21*-nucGFP–positive BPs, they may be neurons which migrate more slowly into the neuronal layer and whose phenotype takes longer to develop. In this context, it should be noted that the present approach of identifying neurons by the appearance of *Tubb3*-mGFP fluorescence does not allow any conclusion as to the specific neuronal subtype.

The speed with which *Tubb3*-mGFP fluorescence became detectable in the newborn neurons after completion of BP mitosis (on average 45 min) is remarkable. In fact, *Tubb3*-mGFP fluorescence was seen to appear in neurites as early as 12 min after mitosis. Appearance of *Tubb3*-mGFP in neurites comprises its transcription, translation, folding to a fluorescent state, membrane insertion and intracellular transport. As it is unlikely that all these steps can occur in a period as short as 12 min, our observations imply that, in the case of BP-derived neurons, the expression of the neuronal phenotype is initiated, at least in some cases, already in the progenitor, for example by inducing neuron-specific genes before mitosis. The previously observed presence of beta-III-tubulin immunoreactivity in some non-surface dividing cells [52] is consistent with this scenario. In this context, the almost three-fold slower appearance of *Tubb3*-mGFP fluorescence in AP-derived than BP-derived neurons presumably reflected a slower onset of transcription of the mGFP mRNA rather than less efficient translation or slower protein maturation. The initiation of the neuronal phenotype already in BPs would be compatible with their lack of cell polarity (in contrast to APs), as will be discussed below.

### Neurogenic as well as non-neurogenic basal progenitors lack apical-basal polarity during mitosis

Apical-basal polarity, notably (i) the maintenance at the apical-most end of the lateral plasma membrane of adherens junctions containing ZO-1 [17], [24], (ii) the concentration of the par3/6/aPKC complex at the apical cell cortex [18], [26], (iii) the presence of an apical plasma membrane bearing a primary cilium [25], [45], [50], and (iv) a cleavage plane parallel to the apical-basal cell axis [4], [14], [21], [22], [39], [41], [51], are thought to be prerequisites for AP daughters to remain APs and re-enter the cell cycle [18]. With regard to BPs, it has previously been shown that these cells retract their apical process prior to mitosis [11]. However, it should be emphasized that it has not previously been addressed, at the level of single cell analysis, whether this retraction is accompanied by a loss of cell polarity in BPs, or whether BPs retain features of apical-basal polarity even after losing their epithelial characteristics. The latter is the case for other delaminated mitotic progenitors, notably the paradigmatic Drosophila neuroblast, which delaminates from an epithelium but retains apical-basal polarity [23].

By using high-resolution imaging of general reporters for plasma membrane and cytosol (membrane GFP, mRFP and MPM-2) that outlined the shape of mitotic BPs, the present data confirm the conclusion [11] that BPs, when in mitosis, lack an apical process that extends all the way to the ventricular surface. We did observe a short apically directed cytoplasmic extension in about a third of the mitotic BPs. However, this is not surprising because the MPM2 epitope is expressed preferentially during the early phases of mitosis, and so we interpret such short extensions as the remnant of the retracting apical process. Despite the existence of this remnant, our analysis of molecular markers of neuroepithelial cell polarity (prominin-1, megalin and ZO-1) demonstrate that essentially all mitotic BPs lack apical-basal polarity. This indicates that loss of apical-basal polarity is indeed a hallmark of the generation of rodent BPs. Our data imply that the previously performed perturbation of apical-basal polarity, which led to an increase in the number of basally dividing cells in the developing mouse neocortex [15], [16], [18], actually mimicked a physiologically occurring transition. It should be emphasized that the available evidence [15], [16], [18], [51], [53], [54], together with the presents results, indicate that loss of apical-basal polarity (including loss of adherens junctions) is necessary, but may not be sufficient, for the generation of progenitors that divide, in an abventricular location, symmetrically into two neurons.

The lack of apical-basal polarity in mitotic BPs that do not express *Tis21*-nucGFP has an important implication with regard to the ability of BP daughters to re-enter the cell cycle. A small proportion (≈10%) of BPs in rodents is thought to generate more BPs (rather than neurons) whose proliferation potential, however, is rather limited [5], [9], [47]. We previously reported that approximately 10% of all BPs are *Tis21*-nucGFP–negative [10]. Assuming that the *Tis21*-nucGFP–negative subpopulation of BPs are the ones that generate more BPs, it follows from their lack of apical-basal polarity that, in contrast to APs, the presence of apical cell constituents during M-phase is not required for BP daughters to re-enter the cell cycle. An interesting question emerging in this context is whether the lack of apical cell constituents in BPs limits their proliferation potential.

### Symmetric divisions of apical and basal progenitors are fundamentally different

Mitotic BPs showed a striking increase in horizontal cleavage planes (i.e. perpendicular to the radial axis of the neuroepithelium), an orientation hardly ever observed for mitotic APs. This presumably reflected a trend towards a randomization of cleavage plane orientation in BPs as compared to APs. Our conclusion in this regard differs from that of a very recent report [48] published after submission of the present study. In APs, the orientation of the cleavage plane parallel to their apical-basal axis acts in concert with the polarized subcellular localization of cell fate determinants along this axis, thereby ensuring their equal distribution to the daughter cells on symmetric division, and a small deviation of the cleavage plane from this orientation suffices for asymmetric division to occur [14], [21], [22]. BPs, in contrast, are fundamentally different in this regard, because they divide symmetrically irrespective of cleavage plane orientation. We suggest this is so because mitotic BPs lack apical-basal polarity, and hence cell fate determinants are unpolarized and distributed equally to the daughter cells with any cleavage plane orientation. For the same reason, such lack of cell polarity would also allow the initiation of the neuronal phenotype already in the progenitor, before the ensuing symmetric cell division.

### In the dorsal telencephalon, *Tis21*-nucGFP–positive apical progenitors generate not only neurons but also basal progenitors

An unexpected finding of the present study was that on time-lapse imaging of the dorsal telencephalon of the double transgenic embryos at the very onset of neurogenesis, surprisingly few (3 of 79) of the *Tis21*-nucGFP–positive APs generated daughter cells showing detectable *Tubb3*-mGFP fluorescence, although tracking was performed on average for almost 5 hours (282 min) and in the longest case for ≈13 hours, i.e. the length of a full cell cycle [55]. Remarkably, when there was detectable *Tubb3*-mGFP expression, it occurred within ≈2 hours after birth of the AP daughter, i.e. well within the average tracking time. Thus, the lack of detectable *Tubb3*-mGFP expression in the vast majority of AP daughters even several hours after their birth is puzzling. Of course, on the one hand, we cannot exclude the possibility that the few *Tubb3*-mGFP–positive AP daughters constituted a minor, specific neuronal subpopulation, and that the typical time course of appearance of a neuronal phenotype (as revealed by *Tubb3*-mGFP expression) in AP daughters takes longer than could be visualized with the present imaging approach (see Results for the reasons that prevented tracking for longer periods). On the other hand, one may wonder why *Tubb3*-mGFP expression was not detectable even in those AP daughters that left the VZ after 5.5 hours (i.e. the average VZ exit time) and in those that could be tracked within the VZ for at least this length of time. If one assumes that upon tracking for longer than 5.5 hours, *Tubb3*-mGFP expression should have become detectable if *Tis21*-nucGFP–positive AP daughters were neurons, alternative possibilities as to the fate of these cells should be considered.

Tis21 expression is an established marker of neurogenic progenitors [10], [27]. Specifically, essentially all neurons have been shown to be derived from *Tis21*-nucGFP–expressing progenitors [10]. This, however, does not necessarily mean that the only type of progeny derived from *Tis21*-nucGFP–expressing APs are neurons. For example, a possibility is that Tis21 expression already occurs in the AP cell cycle preceding the first neurogenic AP cell cycle. However, two considerations render this possibility unlikely. First, if both, (i) *Tis21*-nucGFP–positive but not yet neurogenic APs that generate *Tis21*-nucGFP–positive neurogenic APs, and (ii) *Tis21*-nucGFP–positive APs that generate neurons, would co-exist at similar abundance at E10.5, one would still expect a greater proportion of the *Tis21*-nucGFP–positive apical divisions to generate *Tubb3*-mGFP expressing cells than was actually observed. Second, the time of onset of Tis21 mRNA and protein as well as *Tis21*-nucGFP expression on the one hand, and of the appearance of the first neurons on the other hand, is so similar [10], [27] that it seems incompatible with a scenario in which *Tis21*-nucGFP appearance precedes the appearance of neurons by two cell cycles (about 24 h at the onset of neurogenesis [55]).

**Figure 8:**
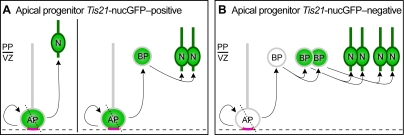
Proposed relationship between *Tis21*-nucGFP–positive and –negative apical progenitors, basal progenitors and neurons in the dorsal telencephalon. (A) Asymmetrically dividing *Tis21*-nucGFP–positive APs. (B) Asymmetrically dividing *Tis21*-nucGFP–negative AP. *Tis21*-nucGFP and *Tubb3*-mGFP are indicated in green; red and dashed line, apical plasma membrane and ventricular surface, respectively; dotted lines, cleavage plane; N, neuron; PP, preplate; VZ, ventricular zone. Note that the proposed model is confined to asymmetrically dividing APs [14] and incorporates observations of the present and previous studies [9], [11].

This leaves us with the most probable explanation that, at the onset of neurogenesis in the dorsal telencephalon of rodents, *Tis21*-nucGFP–positive APs comprise at least two subpopulations, one that generates neurons, and another that generates cells that are committed to the neuronal lineage but are not yet neurons, i.e. BPs (Fig. 8A). This suggests that the heterogeneity of APs observed at later stages of neurogenesis [7], [8] holds true already at the onset the onset of neurogenesis. Our model (Fig. 8) would be in agreement with the previously observed lag of about one day between the appearance of *Tis21*-nucGFP–positive APs and BPs [10]. Moreover, the majority of *Tis21*-nucGFP–positive APs in the telencephalon have been shown to divide asymmetrically in cell biological terms, such that one of the daughters does not inherit apical plasma membrane and adherens junctions [14]. Thus, if the first BPs are generated from *Tis21*-nucGFP–positive APs, this would provide a straightforward explanation why newborn BPs lose apical contact, retract their apical process, and subsequently lack apical-basal polarity at mitosis (Fig. 8A). Another aspect of this model is that the few directly neurogenic divisions of *Tis21*-nucGFP–positive APs that we observed at the very onset of neurogenesis may generate some of the Cajal-Retzius cells (Fig. 8A), a distinct class of neurons that are born at the onset of neurogenesis and that constitute a minor proportion of all cortical neurons [56], [57].

### A unifying concept for the origin of basal progenitors

By analogy, given that *Tis21*-nucGFP–negative BPs also lack apical contact and apical-basal polarity at mitosis, we propose that *Tis21*-nucGFP–negative BPs arise from cell biologically asymmetric divisions of *Tis21*-nucGFP–negative APs (Fig. 8B). Consistent with this, about 10% of the asymmetrically dividing APs are *Tis21*-nucGFP–negative [14], and likewise 10% of all BPs are *Tis21*-nucGFP–negative [10] and divide symmetrically to undergo at least one round of self-amplification before generating neurons [9] (Fig. 8B).

In conclusion, at the onset of neurogenesis, cell biologically asymmetric divisions of APs (i.e. divisions in which only one daughter inherits apical plasma membrane and adherens junctions), irrespective of whether these are *Tis21*-nucGFP–positive or *Tis21*-nucGFP–negative, give rise to cells detached from the lumenal surface of the neural tube. In regions of the rodent CNS other than the telencephalon, most of these cells are neurons, whereas in the telencephalon, most of them are BPs.

## Materials and Methods

### Generation of the *Tubb3*-mGFP and the *Tis21-*nucGFP/*Tubb3-*mGFP transgenic mouse lines

A Bacterial Artificial Chromosome (BAC) (Celera clone ID RP23-214J5) containing the entire beta-III-tubulin open reading frame and including the 50 kb-long 5′ and 3′ flanking regions was used for insertion of the targeting construct (panel A). The targeting construct, containing (from 5′ to 3′) the GAP43 plasma membrane localization signal, 3 myc tag sequences, the EGFP sequence, an SV40 polyadenylation sequence and a hygromycin resistance cassette flanked by loxP recombination sites, was inserted after the first ATG in the first exon of the *beta-III-tubulin* gene (Fig. S1A), using the Red/ET recombinase-based technology. The hygromycin resistance cassette was excised by transferring the modified BAC to a bacterial strain expressing Cre recombinase. The resulting BAC construct was injected into the male pronucleus of 820 fertilized oocytes (strain 129) at a concentration of 2 µg/ml. After 2 hours in vitro development, 20 zygotes were transferred per foster mother (C57Bl/6J). Sixty-five pups were born, two of which were positive by Southern blot using PstI restriction (Fig. S1B) and PCR analyses. One of the two chimeras showed germline transmission, giving rise to green fluorescent pups, and was used as founder to establish the Tg(*beta-III-tubulin*-GAP43-GFP)1Wbh transgenic mouse line (referred to in short as *Tubb3*-mGFP) by crossing with C57Bl/6J mice. The genotype of the mice was assessed by Southern blot, PCR and analysis of embryos/pups obtained after backcrossing with wild type C57Bl/6J mice. The probe for Southern blot analysis corresponded to a major portion (500bp) of the first intron of the *Tubb3* gene (Fig. S1A) and was generated by PCR using digoxygenin-labeled nucleotides and the following oligonucleotide primers: 5′CAATCGGTTCCCCTCAATCAC3′ and 5′TGGCTTCAACACCTGGATGC3′. Genotyping by PCR was performed by checking the presence of GFP using the following oligonucleotide primers: 5′ACCAGCAGAACACCCCCATCG3′ and 5′CGGTCACGAACTCCAGCAGGA3′. After repeated backcrossing with C57Bl/6J mice, *Tubb3*-mGFP homozygous male pups were no longer born, but *Tubb3*-mGFP homozygous female as well as *Tubb3*-mGFP heterozygous male and female pups were obtained at normal Mendelian ratio, were healthy, bred normally, and showed no obvious phenotype. Only *Tubb3*-mGFP heterozygous embryos were used for the experiments.

The *Tubb3*-mGFP transgenic mouse line was crossed with the previously generated *Tis21*-nucGFP knock-in line. Neither mice heterozygous for *Tubb3*-mGFP and homozygous for *Tis21*-nucGFP nor mice heterozygous for both *Tubb3*-mGFP and *Tis21*-nucGFP showed any phenotype; only embryos heterozygous for both *Tubb3*-mGFP and *Tis21*-nucGFP were used for the experiments. Mice were genotyped by PCR; the presence of wild type *Tis21*, *Tis21*-nucGFP and *Tubb3*-mGFP was determined using a cocktail of 4 oligonucleotides: 5′GAGTGGTATGAAAGGCGCAGC3′ (5′ *Tis21*), 5′TTCCAGACCCCGACGTGTGCTCAC3′ (3′ *Tis21*), 5′TCCGCCTGCCTTTTCGTC3′ (5′ *Tubb3*), and 5′GCTCCTCGCCCTTGCTCAC3′ (3′ GFP). To determine whether the *Tis21*-nucGFP/*Tubb3*-mGFP double transgenics were homo- or heterozygous with regard to the *Tubb3*-mGFP transgene, the mice were backcrossed with wild type C57Bl/6J.

The day of the vaginal plug was defined as embryonic day (E) 0.5.

### *In utero* electroporation

*In utero* electroporation of mouse embryos was performed as described [58], except that the topology of the embryos was determined using illumination and a dissecting microscope rather than ultrasound microscopy. Pregnant mice 12 days post coitum were anesthetized with isofluorane vapor and their uteri exposed. Heterozygous E12.5 *Tis21*-nucGFP embryos were injected, through the uterine wall, into the developing telencephalic vesicle with 1–2 µl of an endotoxin-free DNA solution (pCAGGs-GAP43-GFP, pCS2-GAP43-GFP or pCAGGs-mRFP; 0.5–1 µg/µl in PBS) containing a trace of vital dye (Fastgreen), using a glass capillary. Five unidirectional squared electrical pulses (25–30 V, 50 ms each at 1-s intervals) were delivered through platinum electrodes (2 mm diameter, ≈5 mm distance between the electrodes), using a BTX®-ECM®830 electroporator. The orientation of the electric field was used to direct the uptake of the plasmid into cells of the dorsal telencephalon with ventricular contact. After electroporation, the uterus was relocated into the peritoneal cavity and the abdomen sutured. Mice were sacrificed after 18–24 h, and embryos collected for further analysis.

### (Immuno) Fluorescence microscopy on whole-mounts and cryosections

Immunofluorescence was performed according to standard procedures as described [10]. Primary antibodies were: TUJ1, mouse monoclonal antibody, Covance, 1∶200; MPM-2, mouse monoclonal antibody, Upstate, 1∶6000; nestin, mouse monoclonal antibody, USbio, 1∶200; MAP2, mouse monoclonal antibody, Sigma, 1∶1000; Doublecortin, goat polyclonal antibody, Santa Cruz Biotechnology, 1∶200: beta-III-tubulin, mouse monoclonal antibody, Sigma, 1∶300; giantin, mouse monoclonal antibody, Alexis biochemical, 1∶300; prominin-1, rat monoclonal antibody, [25], 1∶300; megalin, rabbit polyclonal antibody, 1∶500 [59]; ZO-1, rabbit polyclonal antibody, Zymed laboratories Inc., 1∶200; aPKC, mouse monoclonal antibody, BD Biosciences, 1∶200. Sections were analyzed either by fluorescence wide-field (Olympus BX61) or confocal (Zeiss Axiovert 200M LSM 510) microscopy.

### Electron microscopy and immunogold labeling

Immunogold EM was performed as described [44], [45]. Sections were labeled with rabbit anti-GFP (1∶100, Invitrogen) followed by 10 nm gold-coupled Protein A, contrasted with a mixture of 1.9% methyl cellulose/0.3% uranyl acetate, and viewed in a Morgagni electron microscope (FEI Company, Eindhoven, NL). Micrographs were taken with a MegaviewII camera and AnalySis software (Olympus/Soft Imaging Systems).

### Two-photon time-lapse video microscopy

Double heterozygous *Tis21*-nucGFP/*Tubb3*-mGFP E9.5-E12.5 embryos, or heterozygous *Tis21*-nucGFP embryos electroporated at E12.5 and allowed to develop for 24 hours, were dissected at room temperature in Dulbecco's PBS (Invitrogen) supplemented with 10% heat-inactivated FCS and 100 U/ml penicillin/streptomycin (Invitrogen). The head without skin (E10.5, E11.5) or the dissected brain (E12.5, E13.5) was immersed in 3% low melting agarose in PBS (Sigma) at 37°C, the sample was cooled to room temperature, and 250–500 µm slices of telencephalon were cut using a manual tissue chopper (Vibratome 600 McIlvain, Ted Pella Inc.), with the orientation of the cutting plane being perpendicular to the A-P axis of the neural tube. Imaging of the dorsal telencephalon was carried out with the X-Y plane being perpendicular to the ventricular surface, and only those slices in which, upon proper orientation in the microscope, most of the radial migration of cells occurred in the focal plane, were selected.

Slices (on average 7) were immersed at room temperature in ≈2.5 ml of type Ia collagen (Cellmatrix, Nitta Gelatin) (diluted to 1.5 mg/ml with DMEM and neutralizing buffer according to the manufacturer's protocol and kept on ice until use), transferred onto a circular 42-mm glass cover slip which constituted the bottom of the incubation chamber of a POC-Chamber-System (Saur, Reutlingen, Germany). After 40 min at 37°C (the time necessary for the collagen to solidify), the slices were cultured at 37°C in 5–7 ml of Neurobasal medium (Invitrogen) supplemented with 10% immediately centrifuged mouse serum (Harlan), 1x N2 supplement (Invitrogen), 1x B27 supplement (Invitrogen) and 100 U/ml penicillin/streptomycin. The POC-Chamber-System was gassed with 40% O_2_, 5% CO_2_, 55% N_2_, with the incubation chamber being sealed with a semi-permeable membrane that allowed gas exchange while preventing evaporation. Slices were superfused with medium (0.1 ml/min) recirculated using a peristaltic pump (Gilson).

Time-lapse analysis was usually started ≈1 h after the beginning of the culture, after visual verification of the integrity of the slice. Images were recorded on an inverted Radiance 2100 two-photon microscope (Bio-Rad) equipped with Nikon optics (ECLIPSE TE300) and a Mira 900 titanium:sapphire laser (Coherent) tuned to 880–900 nm, which was pumped by a Verdi 5 or 10 W solid-state laser (Coherent). A 60x water immersion lens, which was heated to 37°C using an objective heater (Bioptechs), was used to record the GFP fluorescence. Stacks of optical sections, spaced at 2.7 µm intervals, were collected every 8–12 min, starting at 20 µm from the cut surface, yielding optical cuboids with an z-axis of 60–130 µm. Recording was performed using a single scanning run at 512×512 pixel resolution with a scanning speed of typically 166 lines per second. Images were acquired using the Laser 2000 software (BioRad/Zeiss); 3D-reconstructions were computed using Imaris software (Bitplane AG) in the Maximum Intensity Projection rendering mode.

### Analysis of time-lapse microscopy

Twenty-three two-photon time-lapse imaging experiments were carried out on slice cultures of dorsal telencephalon from E10.5 (n = 15), E11.5 (n = 3) and E12.5 (n = 5) *Tis21*-nucGFP/*Tubb3*-mGFP double heterozygous embryos. For each experiment, data were obtained from a single slice, so that each experiment is independent from the other and reflects a separate embryo. Only experiments in which *Tis21*-nucGFP–positive nuclei were observed to undergo interkinetic nuclear migration and mitosis and in which there was no significant apoptosis (as judged by the appearance of pycnotic nuclei) throughout the entire time of acquisition, were considered for analysis.

AP divisions were defined as those occurring at the ventricular surface or at a subapical location (Fig. 4B) [10], whereas BP divisions were defined as those occurring either in the basal region of the VZ (within 3 nuclear diameters from the basal boundary of the VZ), in the emerging neuronal layer adjacent to the VZ, and in the SVZ (when present). 184 mitoses were observed, and the mean±SD of the onset of *Tubb3*-mGFP expression was calculated for AP (n = 3, 127±27 min) and BP (n = 48; 45±23 min) daughters. Those mitoses in which the track of both daughter nuclei was lost before a time corresponding to the mean minus 2x SD of the onset of *Tubb3*-mGFP expression were excluded from the analysis. This resulted in 162 *Tis21*-nucGFP–positive progenitor divisions (n_ E10.5_ = 107, n_ E11.5_ = 25, n_ E12.5_ = 30; 79 APs, 83 BPs) the daughters of which were analyzed.

## Supporting Information

Text S1 (0.05 MB DOC)

Figure S1 The BAC construct used to generate the transgenic Tubb3-mGFP mouse line. (A) The Tubb3-mGFP BAC construct used for male pronuclear injection. Top: beta-III-tubulin (Tubb3) gene contained in the insert of the BAC (Celera ID RP23-214J5). Bottom: construct for insertion into the Tubb3 gene, containing the GAP43 plasma membrane localization signal (striped box), myc tag, (gray box), GFP (black box) and SV40 polyadenylation signal. Length and position of the probe (bar over intron 1) and the position of the PstI restriction sites (arrows) used for Southern analysis are indicated. Scale bar applies to both Tubb3 gene and GFP construct. (B) Southern blot analysis after PstI restriction of wild type (−/−), heterozygous (+/−) and homozygous (+/+) Tubb3-mGFP mice. Arrowhead, Tubb3-mGFP transgene; arrow, endogenous Tubb3 gene.
(0.36 MB TIF)

Figure S2 Intrinsic GFP fluorescence in the central and peripheral nervous system of embryonic and postnatal Tubb3-mGFP mice. (A–C) Whole-mount images of unfixed Tubb3-mGFP embryos at E11.5 (A, dorsal view onto spinal cord (S.C.) and dorsal root ganglia (D.R.G.)), E12.5 (B, limb) and postnatal day 1 (P1) (C, dorsal view onto dissected brain and medulla oblongata). Arrows indicate dorsal root ganglia (A) or telencephalon (C), arrowheads indicate nerve bundles (A, B), asterisk indicates the developing cerebellum (C). Background has been darkened electronically. Scale bars: A and B, 500 µm; C, 3 mm. (D–E) Fluorescence photomicrographs of 12-µm cryosections through E11.5 dorsal root ganglia (D.R.G.) (D) and E14.5 spinal cord (S.C.) (E) of Tubb3-mGFP mouse embryos. Green, intrinsic Tubb3-mGFP fluorescence; blue, Hoechst staining of nuclei (F–K) Fluorescence photomicrographs of 12 µm-thick cryosections through the E11.5 hindbrain of Tubb3-mGFP mice, showing intrinsic Tubb3-mGFP fluorescence (F, H and I, K green) and nestin (G, H red) or MAP2 (J, K red) immunofluorescence; blue, Hoechst staining of nuclei (H, K). Ventricular (apical) surface is down (dashed lines); PP, preplate; NL, neuronal layers. Arrows, dorsal root ganglia (E); arrowheads, nerve bundles (D). Scale bars: A–E, 100 µm; F–K, 20 µm.
(5.66 MB TIF)

Figure S3 Behavior of individual daughter cell pairs arising from apical and basal progenitors. Slice cultures prepared from dorsal telencephalon of E10.5–E12.5 Tis21-nucGFP/Tubb3-mGFP double transgenic mouse embryos were analyzed by two-photon time-lapse video microscopy for the behavior of daughter cells arising from Tis21-nucGFP-expressing APs (A, 79 mitoses) and BPs (B, 83 mitoses) in a total of 23 independent experiments. Bars indicate the length of observation of single daughter cells; black, nucleus remaining in the VZ; white, residence in the VZ of a nucleus that eventually left the VZ (time of exit indicated by right end of white bar); red, residence in the neuronal layer (NL) of a nucleus that exited from the VZ (note that Tubb3-mGFP expression was difficult to discern once a cell had entered the NL); green, nucleus of a daughter cell that eventually expressed Tubb3-mGFP (onset of expression indicated by right end of green bar; tracking was stopped at this time point) (see key in box at bottom). Blue line in (B) indicates the mean exit time of nuclei from the VZ (100 min). Vertical lines to the left of panels A and B indicate the daughter cell pairs used for the quantification of the various classes of behavior summarized in Fig. 5, E and F.
(0.65 MB TIF)

Figure S4 Early polarization events in neurons at the onset of neurogenesis. Slice cultures prepared from dorsal telencephalon of E10.5 (A) and E11.5 (B) Tis21-nucGFP/Tubb3-mGFP double transgenic mouse embryos were analyzed by two-photon video microscopy. (A) Basally-directed relocation of the Golgi complex concomitant with neurite outgrowth but preceding neuronal migration. Open arrows indicate a neuron, identified by Tubb3-mGFP expression, which no longer shows Tis21-nucGFP fluorescence. The cell body resides in the ventricular zone (0–130 min), with bright perinuclear fluorescence in the Golgi complex area (triangles), and grows a process in the basal direction (130–140 min, arrowheads), which branches transiently (180 min). The Golgi complex relocates towards the direction of neuronal migration (230–280 min), prior to migration of the soma towards the neuronal layer (250–370 min). See Supplemental Movie 5. Occasionally, we observed neurons changing the direction of migration, in which case the Golgi complex became positioned towards the future direction of migration before its onset (data not shown). (B) Transient apical neuronal growth cone in the ventricular zone. Fifty-eight cases of neurites growing in the VZ, in the majority of cases with the neuronal cell body migrating within the VZ, were observed. In the example shown, two neurons extend Tubb3-mGFP-positive neurites from the neuronal layer towards the ventricular surface (0–150 min, arrowheads). In half of the 16 cases studied, the neurites reached the apical side of the VZ, and their tips assumed a flattened shape parallel to it. This morphology persisted for up to 90 min (160–250 min, triangles), then the neurite assumed its previous shape and retracted (270 min). The soma of one of the neurons migrates towards the apical surface (230–250 min, open arrows), while growing another process in the basal direction (230–250 min, open triangles), which becomes the leading process as the neuron migrates towards the neuronal layer (250–330 min). Both neurons eventually retract their apical neurites (270–330 min). See Supplemental Movie 6. (A, B) Intrinsic GFP fluorescence is white. Each image is a maximum intensity projection of the single focal planes (2.7-µm steps) that showed the cells of interest. Numbers indicate tracking time (min). Time-lapse intervals: 10 min. Ventricular surface (v) is down and pial surface (p) is up (white bars at left margin). Scale bar, 5 µm.
(2.25 MB TIF)

Movie S1 Two-photon laser scanning time-lapse video microscopy of acute 500 µm-thick slice culture prepared from dorsal telencephalon of E10.5 Tis21-nucGFP/Tubb3-mGFP double transgenic mouse embryos; time-lapse interval 8 min; maximum intensity projection of the single focal planes (2.7-µm steps) that showed the cells of interest. A Tis21-nucGFP-positive neurogenic progenitor divides at the basal side of the ventricular zone. Both daughter cells are neurons as they show Tubb3-mGFP fluorescence in their processes (left daughter, 24–216 min; right daughter, 72–216 min), and enter the neuronal layer. Note the transient apical migration of the left daughter.
(0.53 MB MOV)

Movie S2 Two-photon laser scanning time-lapse video microscopy of acute 500 µm-thick slice culture prepared from dorsal telencephalon of E10.5 Tis21-nucGFP/Tubb3-mGFP double transgenic mouse embryos; time-lapse interval 10 min; maximum intensity projection of the single focal planes (2.7-µm steps) that showed the cells of interest. A Tis21-nucGFP-positive neurogenic progenitor Tis21-nucGFP divides in a subapical location. The nucleus of one of the daughter cells migrates rapidly towards the basal side whereas that of the sibling daughter migrates first to the apical surface and then slowly basally. The cell with the leading nucleus shows Tubb3-mGFP fluorescence in its process (140 min), indicative of it being a neuron; this cell subsequently migrates perpendicular to the X-Y plane of imaging and could not be tracked further.
(0.47 MB MOV)

Movie S3 Two-photon laser scanning time-lapse video microscopy of acute 500 µm-thick slice culture prepared from dorsal telencephalon of E12.5 Tis21-nucGFP/Tubb3-mGFP double transgenic mouse embryos; time-lapse interval 12 min; maximum intensity projection of the single focal planes (2.7-µm steps) that showed the cells of interest. A Tis21-nucGFP-positive neurogenic progenitor migrates rapidly from the basal boundary of the ventricular zone to the apical surface and divides. The nuclei of the daughter cells migrate towards the basal side, with one nucleus leading . The leading nucleus was tracked until it entered the neuronal layer, whereas the trailing nucleus was tracked until it disappeared due to photobleaching.
(0.90 MB MOV)

Movie S4 Two-photon laser scanning time-lapse video microscopy of acute 500 µm-thick slice culture prepared from dorsal telencephalon of E13.5 eterozygous Tis21-nucGFP transgenic mouse embryos, electroporated in utero at E12.5 with GAP43GFP. Time-lapse interval 10 min; maximum intensity projection of the single focal planes (2.5-µm steps) that showed the cells of interest. An electroporated cell on the basal side of the neuroepithelium (BP), retracts its apical contact (red arrowhead) prior to mitosis. Daughters originating from such BP have a neuron-like morphology.
(1.53 MB MOV)

Movie S5 Two-photon laser scanning time-lapse video microscopy of acute 500 µm-thick slice culture prepared from dorsal telencephalon of E10.5 Tis21-nucGFP/Tubb3-mGFP double transgenic mouse embryos, time-lapse interval 10 min; maximum intensity projection of the single focal planes (2.7-µm steps) that showed the cells of interest. A Tubb3-mGFP-positive neuron, which no longer shows Tis21-nucGFP fluorescence, resides in the ventricular zone, with bright perinuclear fluorescence in the Golgi complex area, and grows a process in the basal direction, which branches transiently. The Golgi complex relocates towards the direction of neuronal migration, prior to migration of the soma towards the neuronal layer.
(0.60 MB MOV)

Movie S6 Two-photon laser scanning time-lapse video microscopy of acute 500 µm-thick slice culture prepared from dorsal telencephalon of E11.5 Tis21-nucGFP/Tubb3-mGFP double transgenic mouse embryos, Time-lapse interval: 10 min; maximum intensity projection of the single focal planes (2.7-µm steps) that showed the cells of interest. The processes of two Tubb3-mGFP-positive neurons grow towards the ventricular surface and flatten. The soma of one of the neurons migrates towards the apical surface, while growing another process in the basal direction, which becomes the leading process as the neuron migrates towards the neuronal layer. Both neurons eventually retract their apical neurites.
(2.09 MB MOV)
