# Supplementary material for: Live Imaging at the Onset of Cortical Neurogenesis Reveals Differential Appearance of the Neuronal Phenotype in Apical versus Basal Progenitor Progeny
Source: PLoS One. 2008 Jun 11;3(6):e2388. doi: 10.1371/journal.pone.0002388 (PMC2398773; doi:10.1371/journal.pone.0002388)
Supplement: Text S1 — (0.05 MB DOC) [file pone.0002388.s001.doc]

**Text S1**

Accompanying Text to Figure S4

Neurons displayed a characteristic sequence of events: extension of uni- or multi-polar processes without movement of the soma, positioning of the Golgi complex towards the future direction of migration, and finally migration of the soma (Fig. S4A and Movie S5). Occasionally, we observed neurons changing the direction of migration, in which case the Golgi complex became positioned towards the future direction of migration before its onset (data not shown). Likewise, the association of *Tubb3*-mGFP fluorescence with the plasma membrane allowed monitoring specific aspects of neurite outgrowth, which is intimately linked to neuronal polarization. Thus, we observed neurons extending *Tubb3*-mGFP–positive neurites from the neuronal layer towards the ventricular surface n some cases reaching the apical side of the VZ, and their tips assumed a flattened shape parallel to it. Occasionally, neurite extension towards the ventricular surface was followed by migration of the soma, which subsequently returned to the neuronal layer (Fig. S4B and Movie S6).
