# Supplementary figures and images for: Live Imaging at the Onset of Cortical Neurogenesis Reveals Differential Appearance of the Neuronal Phenotype in Apical versus Basal Progenitor Progeny
Source: PLoS One. 2008 Jun 11;3(6):e2388. doi: 10.1371/journal.pone.0002388 (PMC2398773; doi:10.1371/journal.pone.0002388)

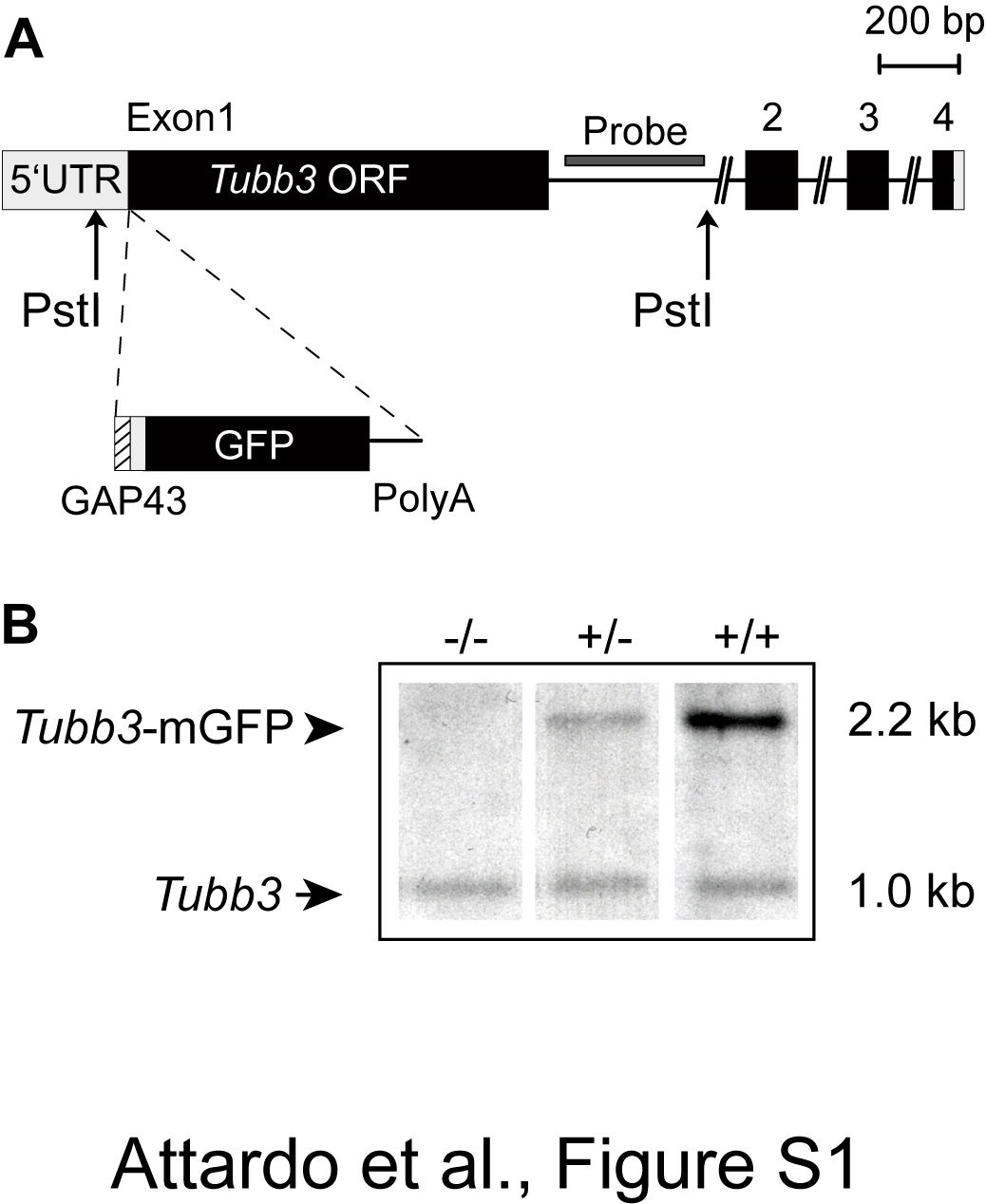

Supplement: Figure S1 — The BAC construct used to generate the transgenic Tubb3-mGFP mouse line. (A) The Tubb3-mGFP BAC construct used for male pronuclear injection. Top: beta-III-tubulin (Tubb3) gene contained in the insert of the BAC (Celera ID RP23-214J5). Bottom: construct for insertion into the Tubb3 gene, containing the GAP43 plasma membrane localization signal (striped box), myc tag, (gray box), GFP (black box) and SV40 polyadenylation signal. Length and position of the probe (bar over intron 1) and the position of the PstI restriction sites (arrows) used for Southern analysis are indicated. Scale bar applies to both Tubb3 gene and GFP construct. (B) Southern blot analysis after PstI restriction of wild type (−/−), heterozygous (+/−) and homozygous (+/+) Tubb3-mGFP mice. Arrowhead, Tubb3-mGFP transgene; arrow, endogenous Tubb3 gene. (0.36 MB TIF) [file pone.0002388.s002.tif]

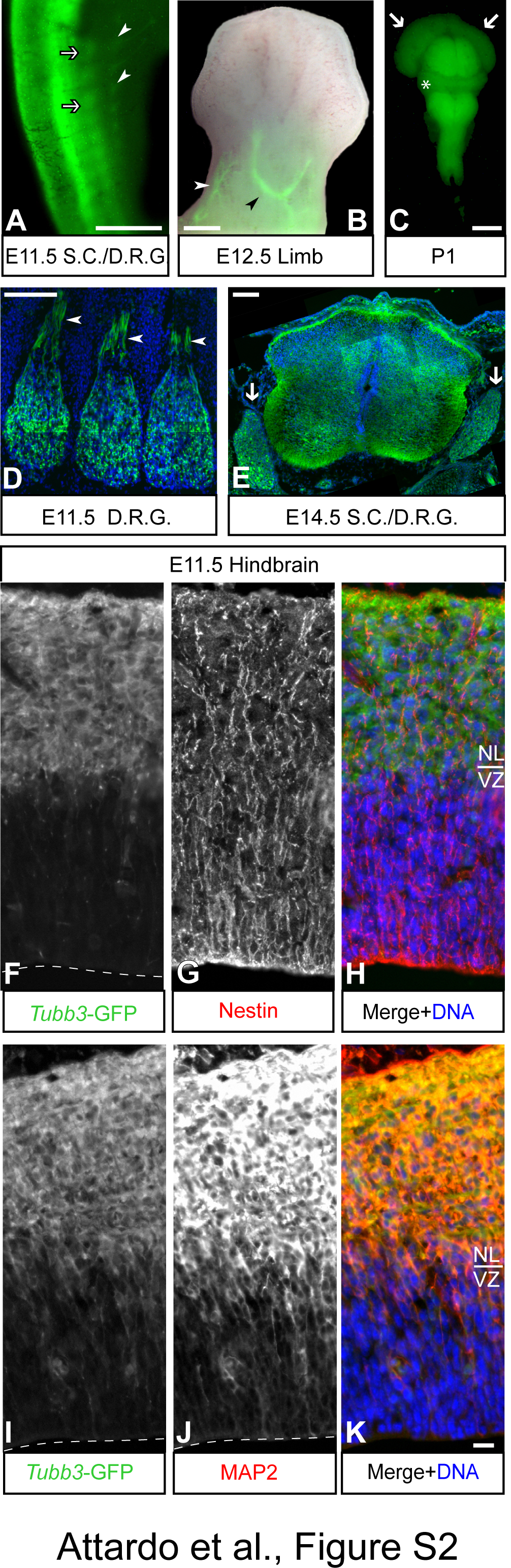

Supplement: Figure S2 — Intrinsic GFP fluorescence in the central and peripheral nervous system of embryonic and postnatal Tubb3-mGFP mice. (A–C) Whole-mount images of unfixed Tubb3-mGFP embryos at E11.5 (A, dorsal view onto spinal cord (S.C.) and dorsal root ganglia (D.R.G.)), E12.5 (B, limb) and postnatal day 1 (P1) (C, dorsal view onto dissected brain and medulla oblongata). Arrows indicate dorsal root ganglia (A) or telencephalon (C), arrowheads indicate nerve bundles (A, B), asterisk indicates the developing cerebellum (C). Background has been darkened electronically. Scale bars: A and B, 500 µm; C, 3 mm. (D–E) Fluorescence photomicrographs of 12-µm cryosections through E11.5 dorsal root ganglia (D.R.G.) (D) and E14.5 spinal cord (S.C.) (E) of Tubb3-mGFP mouse embryos. Green, intrinsic Tubb3-mGFP fluorescence; blue, Hoechst staining of nuclei (F–K) Fluorescence photomicrographs of 12 µm-thick cryosections through the E11.5 hindbrain of Tubb3-mGFP mice, showing intrinsic Tubb3-mGFP fluorescence (F, H and I, K green) and nestin (G, H red) or MAP2 (J, K red) immunofluorescence; blue, Hoechst staining of nuclei (H, K). Ventricular (apical) surface is down (dashed lines); PP, preplate; NL, neuronal layers. Arrows, dorsal root ganglia (E); arrowheads, nerve bundles (D). Scale bars: A–E, 100 µm; F–K, 20 µm. (5.66 MB TIF) [file pone.0002388.s003.tif]

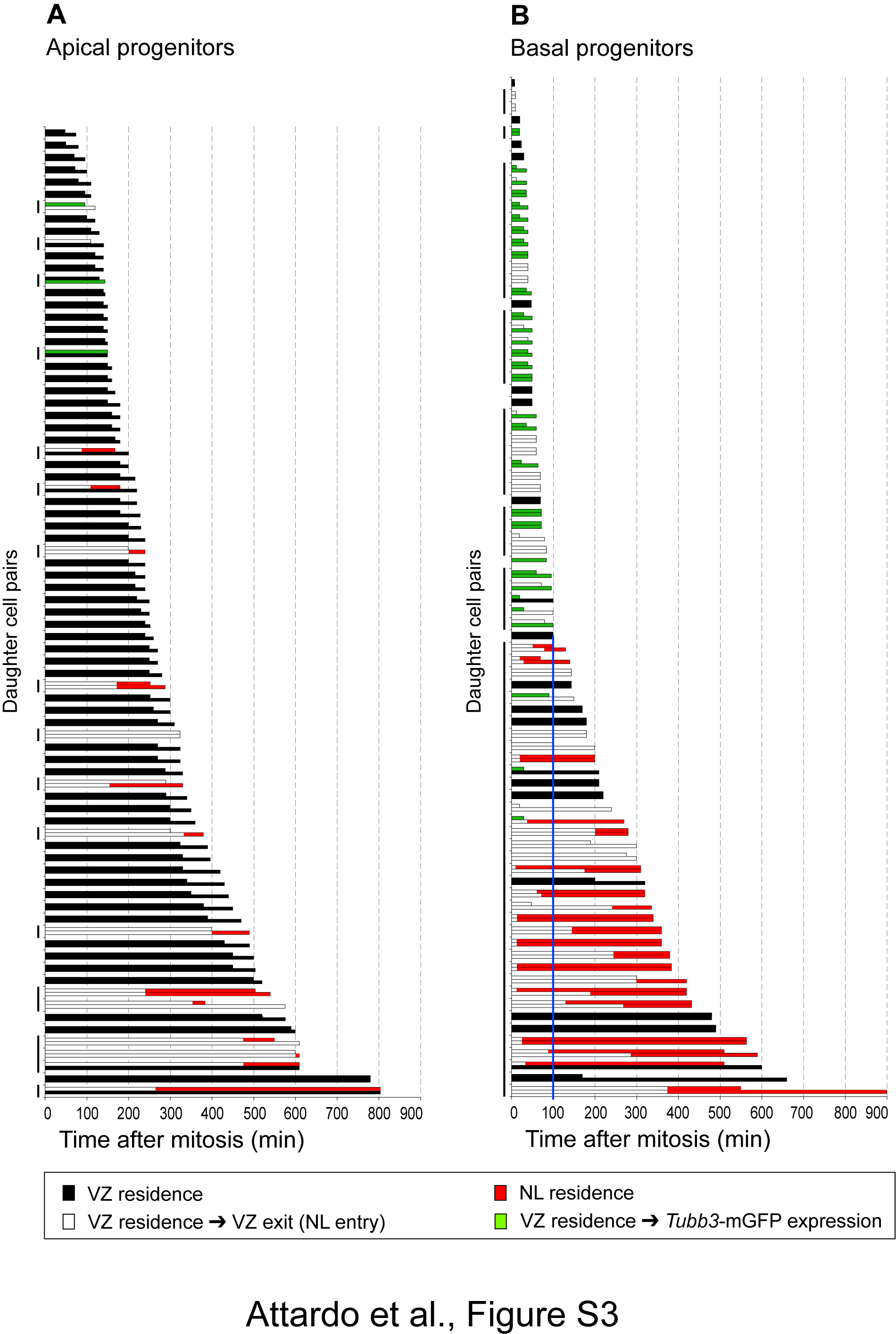

Supplement: Figure S3 — Behavior of individual daughter cell pairs arising from apical and basal progenitors. Slice cultures prepared from dorsal telencephalon of E10.5–E12.5 Tis21-nucGFP/Tubb3-mGFP double transgenic mouse embryos were analyzed by two-photon time-lapse video microscopy for the behavior of daughter cells arising from Tis21-nucGFP-expressing APs (A, 79 mitoses) and BPs (B, 83 mitoses) in a total of 23 independent experiments. Bars indicate the length of observation of single daughter cells; black, nucleus remaining in the VZ; white, residence in the VZ of a nucleus that eventually left the VZ (time of exit indicated by right end of white bar); red, residence in the neuronal layer (NL) of a nucleus that exited from the VZ (note that Tubb3-mGFP expression was difficult to discern once a cell had entered the NL); green, nucleus of a daughter cell that eventually expressed Tubb3-mGFP (onset of expression indicated by right end of green bar; tracking was stopped at this time point) (see key in box at bottom). Blue line in (B) indicates the mean exit time of nuclei from the VZ (100 min). Vertical lines to the left of panels A and B indicate the daughter cell pairs used for the quantification of the various classes of behavior summarized in Fig. 5, E and F. (0.65 MB TIF) [file pone.0002388.s004.tif]

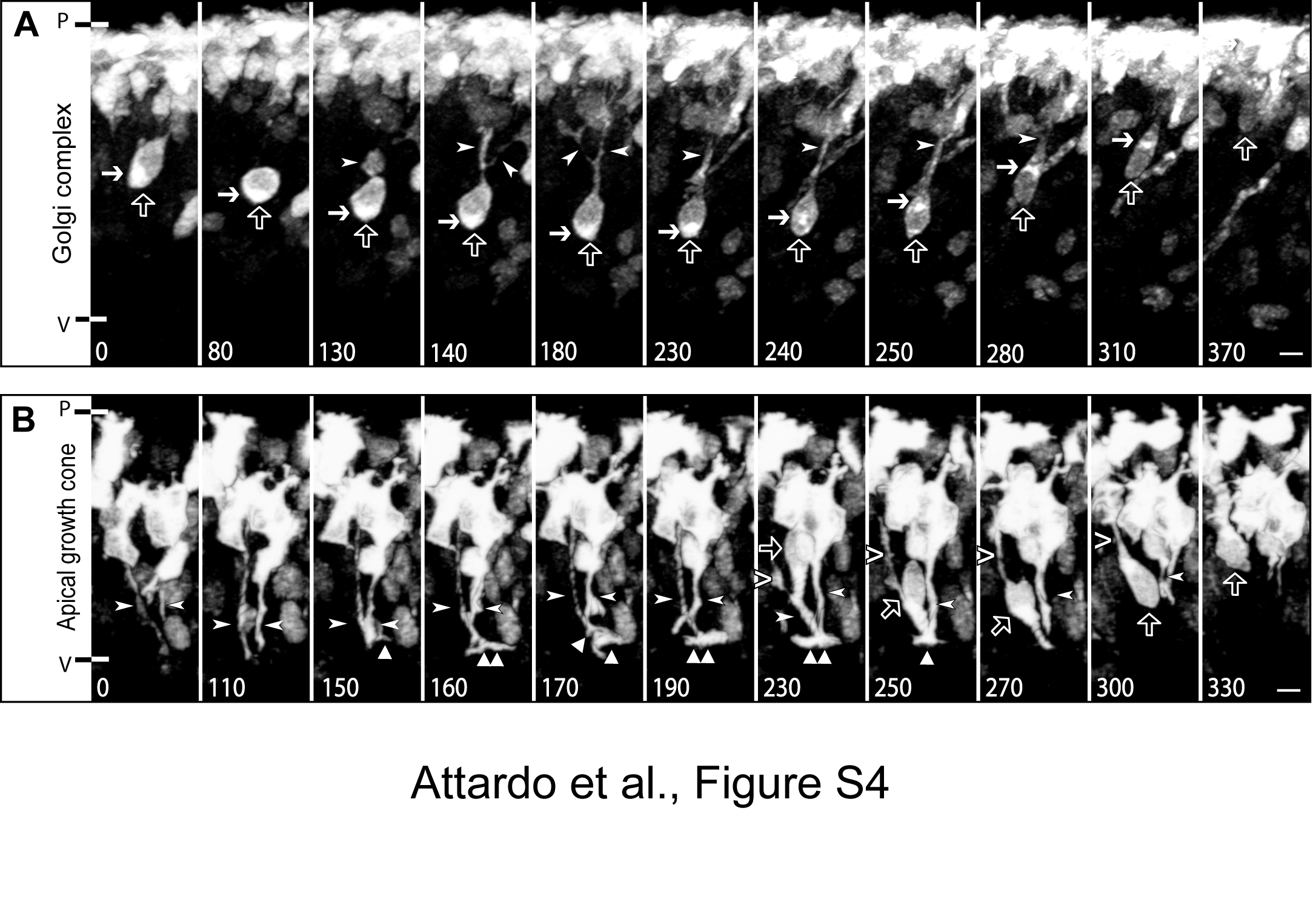

Supplement: Figure S4 — Early polarization events in neurons at the onset of neurogenesis. Slice cultures prepared from dorsal telencephalon of E10.5 (A) and E11.5 (B) Tis21-nucGFP/Tubb3-mGFP double transgenic mouse embryos were analyzed by two-photon video microscopy. (A) Basally-directed relocation of the Golgi complex concomitant with neurite outgrowth but preceding neuronal migration. Open arrows indicate a neuron, identified by Tubb3-mGFP expression, which no longer shows Tis21-nucGFP fluorescence. The cell body resides in the ventricular zone (0–130 min), with bright perinuclear fluorescence in the Golgi complex area (triangles), and grows a process in the basal direction (130–140 min, arrowheads), which branches transiently (180 min). The Golgi complex relocates towards the direction of neuronal migration (230–280 min), prior to migration of the soma towards the neuronal layer (250–370 min). See Supplemental Movie 5. Occasionally, we observed neurons changing the direction of migration, in which case the Golgi complex became positioned towards the future direction of migration before its onset (data not shown). (B) Transient apical neuronal growth cone in the ventricular zone. Fifty-eight cases of neurites growing in the VZ, in the majority of cases with the neuronal cell body migrating within the VZ, were observed. In the example shown, two neurons extend Tubb3-mGFP-positive neurites from the neuronal layer towards the ventricular surface (0–150 min, arrowheads). In half of the 16 cases studied, the neurites reached the apical side of the VZ, and their tips assumed a flattened shape parallel to it. This morphology persisted for up to 90 min (160–250 min, triangles), then the neurite assumed its previous shape and retracted (270 min). The soma of one of the neurons migrates towards the apical surface (230–250 min, open arrows), while growing another process in the basal direction (230–250 min, open triangles), which becomes the leading process as the neuron migrates towards the neur [file pone.0002388.s005.tif]
